# Structural and functional characterization of a metagenomically derived γ‐type carbonic anhydrase and its engineering into a hyperthermostable esterase

**DOI:** 10.1002/pro.70396

**Published:** 2025-11-26

**Authors:** Charoutioun S. Bodourian, Mohsin Imran, Nikolaos D. Georgakis, Anastassios C. Papageorgiou, Nikolaos E. Labrou

**Affiliations:** ^1^ Laboratory of Enzyme Technology, Department of Biotechnology School of Applied Biology and Biotechnology, Agricultural University of Athens Athens Greece; ^2^ Turku Bioscience Centre University of Turku and Åbo Akademi University Turku Finland

**Keywords:** carbon dioxide, carbonic anhydrase, crystallography, hyperthermophiles, metagenomics analysis, thermostability

## Abstract

The 16S microbial community profiling of a metagenomics library from geothermal spring at Lisvori (Lesvos island, Greece) enabled the identification of a putative sequence exhibiting 95% identity to the γ‐type carbonic anhydrase (γ‐CA) from *Caloramator australicus* (γ‐*Ca*CA). The sequence of γ‐*Ca*CA was amplified by PCR, cloned, and expressed in *E. coli*. Activity assays showed that γ‐*Ca*CA possesses very low, but detectable, anhydrase activity, while exhibiting no measurable esterase activity. Differential scanning fluorimetry (DSF) revealed that the enzyme shows high thermal stability with a melting temperature (*T*_*m*_) approximately 65–75°C in the pH range between 5.5 and 9.0. The structure of γ*‐Ca*CA was determined by X‐ray crystallography at 1.11 Å resolution, the highest resolution reported so far for a γ*‐*CA. The enzyme was crystallized as a trimer in the crystallographic asymmetric unit and contains three zinc‐binding sites, one at each interface of neighboring subunits of the trimer. Structure‐based rational design enabled the design and creation of a mutant enzyme (γ*‐Ca*CAmut) which possessed a heptapeptide insertion at the active‐site loop and two‐point mutations. Kinetic analysis demonstrated that γ‐*Ca*CAmut was successfully converted into a catalytically active esterase indicating successful activity gain through structure‐guided engineering. The thermostability of γ‐*Ca*CAmut was significantly increased, aligning with the thermostability typically observed in hyperthermostable enzymes. X‐ray crystallographic analysis of the γ‐*Ca*CAmut structure at 2.1 Å resolution, provided detailed structural insights into how the mutations impact the overall enzyme structure, function, and thermostability. These findings provide valuable structural and functional insights into γ‐CAs and demonstrate a strategy for converting an inactive enzyme into a catalytically active form through rational design.

**Abbreviations**
*Bps*CA: CA from *Burkholderia pseudomallei*
Cag: CA from *Clostridioides difficile*
Cam: CA from *Methanosarcina thermophila*
*Cd*CA: CA from *Thalassiosira weissflogii*
IPTG: Isopropyl‐β‐D‐thiogalactopyranoside
PAGE: polyacryamide gel electrophoresis
RicA: CA from *Brucella abortus*
SDS: sodium dodecyl sulfate
*Tt*CA: CA from *Thermus thermophilus* HB8
YrdA: CA from *E. coli*

## 1 INTRODUCTION

CAs are metalloenzymes that catalyze the reversible hydration of carbon dioxide to bicarbonates according to the reaction *CO*_2_ + *H*_2_ *O* ↔ *HCO*_3_^−^ + *H* ^+^. They are ubiquitous enzymes involved in fundamental processes like photosynthesis, respiration, pH homeostasis, and ion transport (Supuran, 2016a).

CAs are widespread in nature and are classified as α, β, γ, δ, ζ, η, θ and ι CAs (Aspatwar et al., 2014; Capasso & Supuran, 2015, 2024; Hirakawa et al., 2022; Nocentini et al., 2021; Parisi et al., 2000). CAs from mammalian sources are grouped as α‐CAs, whereas CAs from plants, algae and eubacteria are grouped as β‐CAs (Ferraroni, 2024; Hirakawa et al., 2022; Ludwig, 2016). γ‐CAs are found in archaea and eubacteria (Angeli, 2024). The η‐, θ‐, and ι‐families have recently been discovered (Nocentini et al., 2021). CAs from marine diatoms and algae are grouped as δ, ζ, and ι (Nocentini et al., 2021). η‐CA was identified in *Plasmodium* spp. (Del Prete et al., 2014) and ι‐CA has been discovered in diatom *Thalassiosira pseudonana* and in the genome of *Burkholderia territorii* (Capasso & Supuran, 2024). There is no significant sequence identity or structural similarity between the CA families. Notably, CAs display impressive instances of convergent evolution, lacking substantial sequence or structural resemblances, indicating that they do not have a shared ancestor (Capasso & Supuran, 2024; Hirakawa et al., 2022; Nocentini et al., 2021).

CAs are not only highly effective catalysts for the interconversion between carbon dioxide/bicarbonate but also display catalytic versatility, participating in several other hydrolytic processes (Capasso & Supuran, 2024; Supuran, 2016a, 2023). All CA families use metal hydroxide nucleophilic species and possess a unique active site architecture, with half of it hydrophilic and the opposing part hydrophobic, allowing these enzymes to act as some of the most effective catalysts known in nature (Supuran, 2016a). The active site of CAs generally contains a zinc‐binding site. However, other ions such as Mg^2+^, Ni^2+^, Fe^2+^, and Cd^2+^ have also been found at the active sites of CAs (Alterio et al., 2015; Lionetto et al., 2016; Macauley et al., 2009). Recently, scientists (Nocentini et al., 2021) identified in microalgae *Bigelowiella natans* and *Anabaena* sp. PCC7120 catalytically active ι‐CAs without a metal ion cofactor.

Members of the γ‐type CAs (γ‐CAs) family have mainly been isolated from methane‐producing bacteria that grow in hot springs. γ‐CAs are cytoplasmic proteins and are crucial for various functions, such as regulating internal pH and aiding CO_2_ transportation for photosynthesis, highlighting the overall significance of these enzymes.

γ‐CAs belong to a diverse superfamily of proteins that share the left‐handed parallel beta‐helix (LbetaH) motif (Kisker et al., 1996; Parisi et al., 2000). Functional γ‐CAs were found to be homotrimers, containing identical monomers with repeating hexapeptides that are essential for the formation of the LbetaH motif (Capasso & Supuran, 2024; Hirakawa et al., 2022). Their active sites are located between monomer interfaces, demonstrating convergent evolution in response to comparable catalytic requirements in various enzyme classes (Fu et al., 2008).

Geothermal water and soil are challenging natural environments that can be a valuable source of genetic resources regarding the microbial diversity of species present, including thermophilic and hyperthermophilic microorganisms (Arbab et al., 2022). Metagenomics enables scientists to obtain genetic resources for novel proteins, enzymes, and metabolites from organisms that are not cultivable, allowing for the direct assessment of huge genetic diversity within natural microbial communities (Williams et al., 2024). Enzymes from natural thermophilic and hyperthermophilic microorganisms are more active and stable than those from mesophilic microorganisms of plant and animal origin and are therefore considered valuable tools in industrial biotechnology (Mesbah, 2022).

The intense volcanic activity that occurred on Lesvos Island, Greece, during the Miocene (18.5–17.0 Ma) led to the creation of volcanic springs in Lisvori with a temperature of 70°C (Lambrakis et al., 2013). To the best of our knowledge, the microbial ecosystems of these geothermal springs have not received much attention so far. In the present study, metagenomics analysis of soil samples from Lisvori's geothermal spring enabled the identification of a novel thermostable and nearly inactive γ‐CA with 95% sequence identity to a *Caloramator australicus* γ‐CA (γ‐*Ca*CA). The aim of this study was to use structure‐guided engineering to convert the nearly inactive γ‐*Ca*CA scaffold into a biocatalyst with enhanced CO₂ hydration and esterase activities. Structure‐based rational engineering of γ‐*Ca*CA led to the creation of a hyperthermostable enzyme variant (γ‐*Ca*CAmut) with esterase activity, providing an ideal metalloenzyme for biocatalytic applications at high temperatures.

## 2 RESULTS AND DISCUSSION

### 2.1 Composition of the microbial community at Lisvori hot spring

**FIGURE 1:**
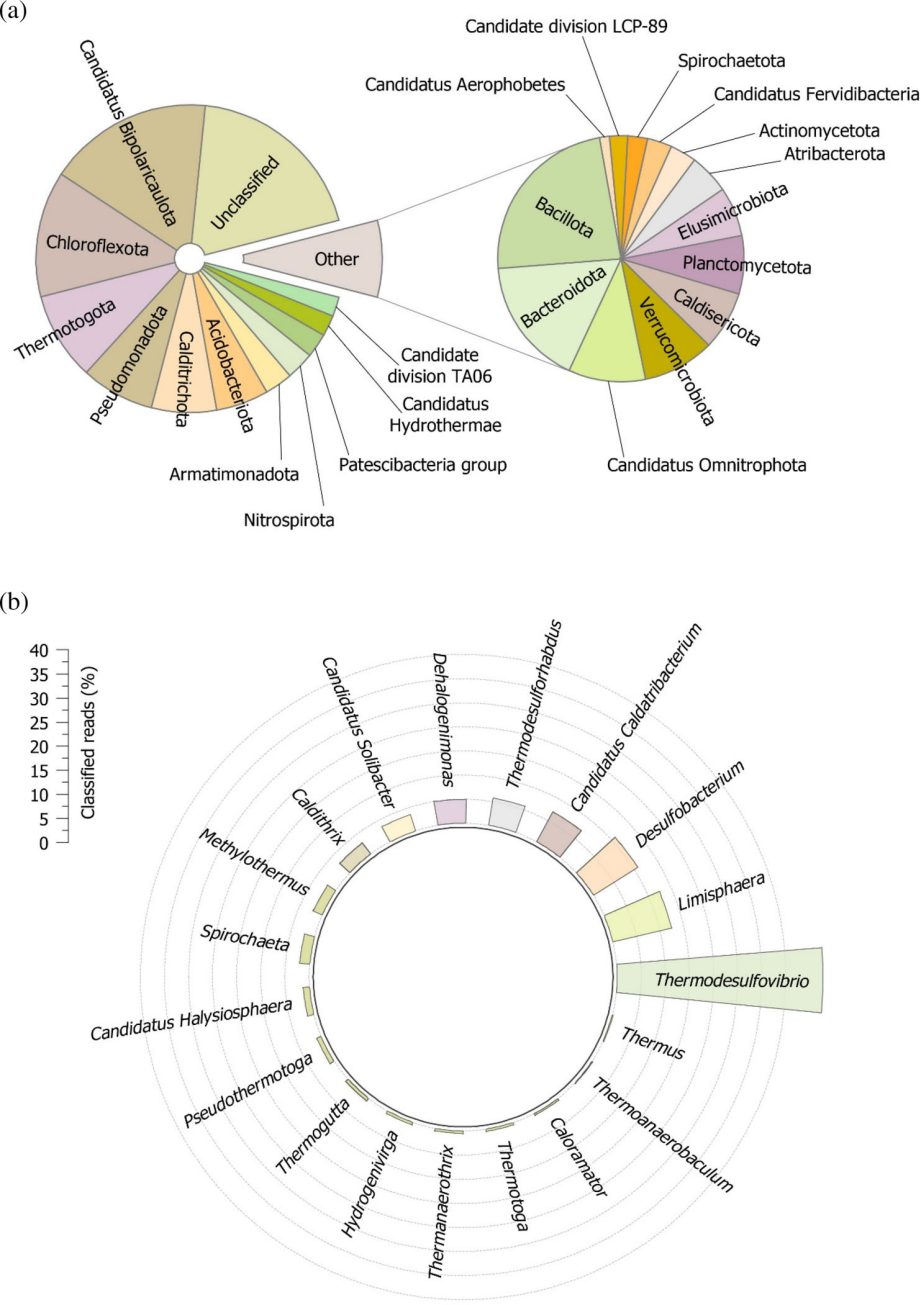
(a) Representative phyla of the microbial communities of the geothermal spring at Lisvori (Lesvos inland, Greece), following classification of the V3‐V4 hypervariable region of the 16S rRNA gene. (b) Representative bacterial genera that were identified through the analysis of the bacterial communities of the hot spring. The depicted genera correspond to approximately 6% of the total reads initially acquired during the sequencing of the V3‐V4 hypervariable region of the 16S rRNA gene.

The 16S rRNA gene is an adequate target for studying bacterial evolution and ecology, facilitating further analysis of phylogenetic relationships among different taxa as it is highly conserved among prokaryotes (Liu et al., 2024), (Regueira‐Iglesias et al., 2023). In the present study, the V3‐V4 regions of the 16S rRNA gene were selected to analyze the microbial community of the Lisvori hot spring. Hot springs serve as natural laboratories for investigating microbial adaptation to extreme environments, offering a valuable genetic reservoir of unique enzymes that drive remarkable evolutionary traits (Burkhardt et al., 2023; Giovannelli et al., 2022). Analysis of the results obtained revealed 214 amplicon sequence variants (ASVs) associated with bacteria. A group of 22 ASVs was categorized as “unclassified” due to a lack of further taxonomic detail beyond the Domain/Kingdom level, matching 18.3% of the total sample reads. Figure 1a displays the representative phyla constituting the microbial community of the hot spring, with a total of 30 bacterial phyla identified as contributing to the detected population at the sampling site. Figure 1b, in turn, depicts the thermophilic bacterial genera that were identified with high confidence, based on sequence similarities exceeding 97%.

According to the analysis of the obtained results, the hot spring in Lisvori constitutes a thriving microbial community in terms of diversity. The biodiversity of the hot spring is affected not only by the increased temperature of the sampling site (approximately 70°C) but also by site‐specific biochemical and environmental factors (Podar et al., 2020). Approximately 40% of the total bacterial ASVs belong to Phylum Candidatus Bipolaricaulota (17.3%), Chloroflexota (13.2%) and Thermotogota (9.2%), while the next most abundant phylum is Pseudomonadota (7.7%). These predominant phyla are accompanied by less abundant phyla such as Calditrichota, Acidobacteriota, Armatimonadota, and Nitrospirota. Interestingly, almost 20% of the total bacterial ASVs could not be classified to any known Phylum. As shown in Figure 1b, bacteria belonging to the genera *Thermodesulfovibrio*, *Limisphaera*, *Desulfobacterium*, *Candidatus* *Caldatribacterium*, and *Thermodesulforhabdus* appear to represent more than 70% of the total classified sequences up to the genus level.

Among the identified thermophilic species, *Caloramator australicus* is one of the least characterized organisms. It is a Gram‐positive thermo‐anaerobe of the phylum Bacillota (Ogg & Patel, 2009) and its ~2.65‐Mb genome has been sequenced (Ogg & Patel, 2011). Interest in *C. australicus* comes from its ability to utilize metals as terminal electron acceptors, which may result in its colonization and promotion of metal corrosion. The metal corrosion capability of *C. australicus* shows notable promise for the development of microbial fuel cell technology (Fu et al., 2013).

BLAST search of *C. australicus* genome (Accession number: NZ_CAKP01000082.1) using as a query the sequence of the γ‐CA from *Methanosarcina thermophila* (UniProt accession number P40881) revealed the presence of a single gene (GenBank: CCC57716.1) homolog to the γ‐type CAs (γ‐*Ca*CA). γ‐*Ca*CA is composed of 167 amino acids with a molecular mass of 18.3 kDa and theoretical isoelectric point (pI) of 5.87.

### 2.2 Cloning, expression, purification and activity assessment of γ*‐* *Ca*CA

PCR was used to amplify the full‐length γ‐*Ca*CA sequence from the eDNA sample. The resulting PCR amplicon was cloned into the T7 expression vector pETite™ C‐His vector. This plasmid was used to transform the *E. coli* Rosetta™ 2(DE3)pLysS cells as the expression host. The recombinant enzyme was expressed at remarkably high levels, facilitating its subsequent purification and characterization (Figure S1a). The 6xHis residues tagged at the C‐terminus of the recombinant enzyme enabled rapid purification by immobilized metal ion affinity chromatography on a Ni^2+^‐IDA‐Sepharose affinity column. The purity of the final γ‐*Ca*CA enzyme was evaluated by SDS‐PAGE (Figure S1a).

The catalytic activity of the purified enzyme was assessed towards the two model reactions that are naturally catalyzed by CAs: the CO_2_ hydration and the hydrolysis of p‐NPA ester. The enzyme, under a range of different pH (5–10) and temperature (10–60°C) conditions, did not show measurable anhydrase or esterase activity, in agreement with other γ‐type CA homologs (Fu et al., 2013; Ogg & Patel, 2011; Wang et al., 2019). However, analysis of anhydrase activity using stopped‐flow instrumentation allowed a marginal detection of low anhydrase activity (see Figure S2).

### 2.3 Thermostability analysis by differential scanning fluorimetry

**FIGURE 2:**
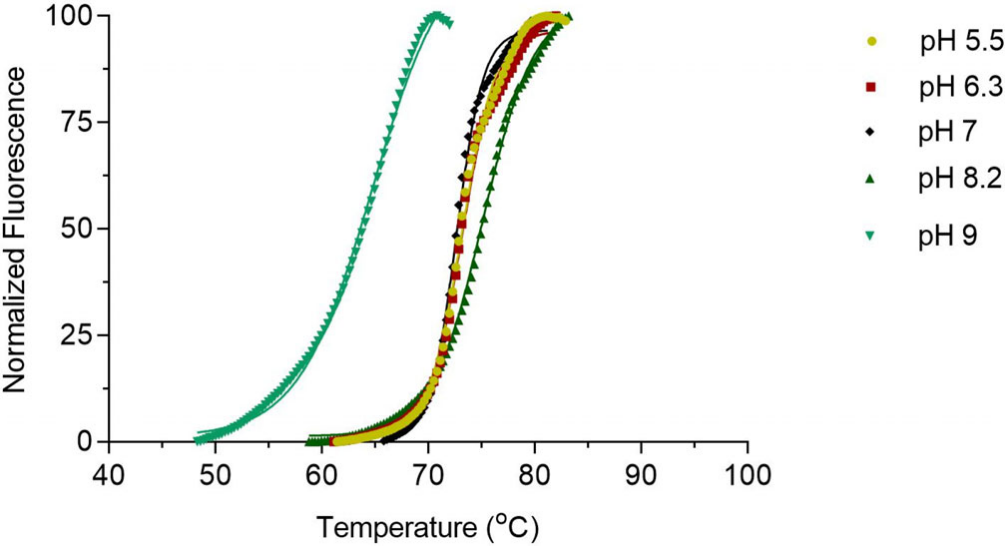
DSF for the determination of melting temperatures (*T*_*m*_) at different pH values (5.5–9.0). The figure depicts normalized denaturation curves for γ*‐Ca*CA. DSF was carried out in buffers with different pH values: 5.5 (●), 6.3 (■), 7(♦), 8.2 (▲) and 9.0 (▼). Each normalized curve represents the mean value of three independent experiments. Curves were fit with the Boltzmann sigmoidal model (solid lines). Goodness of fit (*R* ^2^) values for each pH are 0.99, 0.98, 0.98, 0.99, and 0.98, respectively.

The thermal stability of the γ*‐Ca*CA was assessed by employing DSF (Figure 2) and *T*_*m*_ values (Table 1) were determined across a range of pH conditions. γ‐*Ca*CA exhibited *T*_*m*_ values ranging from 64°C to 75°C across a pH range of 5.5 to 9.0, indicating its thermostable nature. Its high stability over a wide pH range underscores its potential utility in biotech applications that demand both high thermal resistance and tolerance to pH fluctuations. The thermostability of CAs from thermophilic organisms has been documented in the literature. For example, an α‐CA from *Sulfurihydrogenibium yellowstonense* demonstrated a *T*_*m*_ of approximately 90°C (Fredslund et al., 2018). Similarly, a β‐class CA from *Methanobacterium thermoautotrophicum* was reported to maintain stability up to 75°C (Smith & Ferry, 1999).

**TABLE 1:** Melting temperatures (*T*_*m*_) of γ*‐Ca*CA and γ*‐Ca*CAmut measured at different pH values using DSF.

γ*‐Ca*CA		γ*‐Ca*CAmut	
pH	*T*_*m*_	pH	*T*_*m*_
5.5	73.2 ± 0.1	5.0	60.0 ± 0.19
6.3	73.2 ± 0.1	6.0	72.8 ± 0.1
7.0	72.5 ± 0.1	7.0	90.0 ± 0.5
8.2	75.2 ± 0.1		
9.0	64.7 ± 0.2		

### 2.4 Crystallization and structure analysis of γ*‐* *Ca*CA by x‐ray crystallography

**FIGURE 3:**
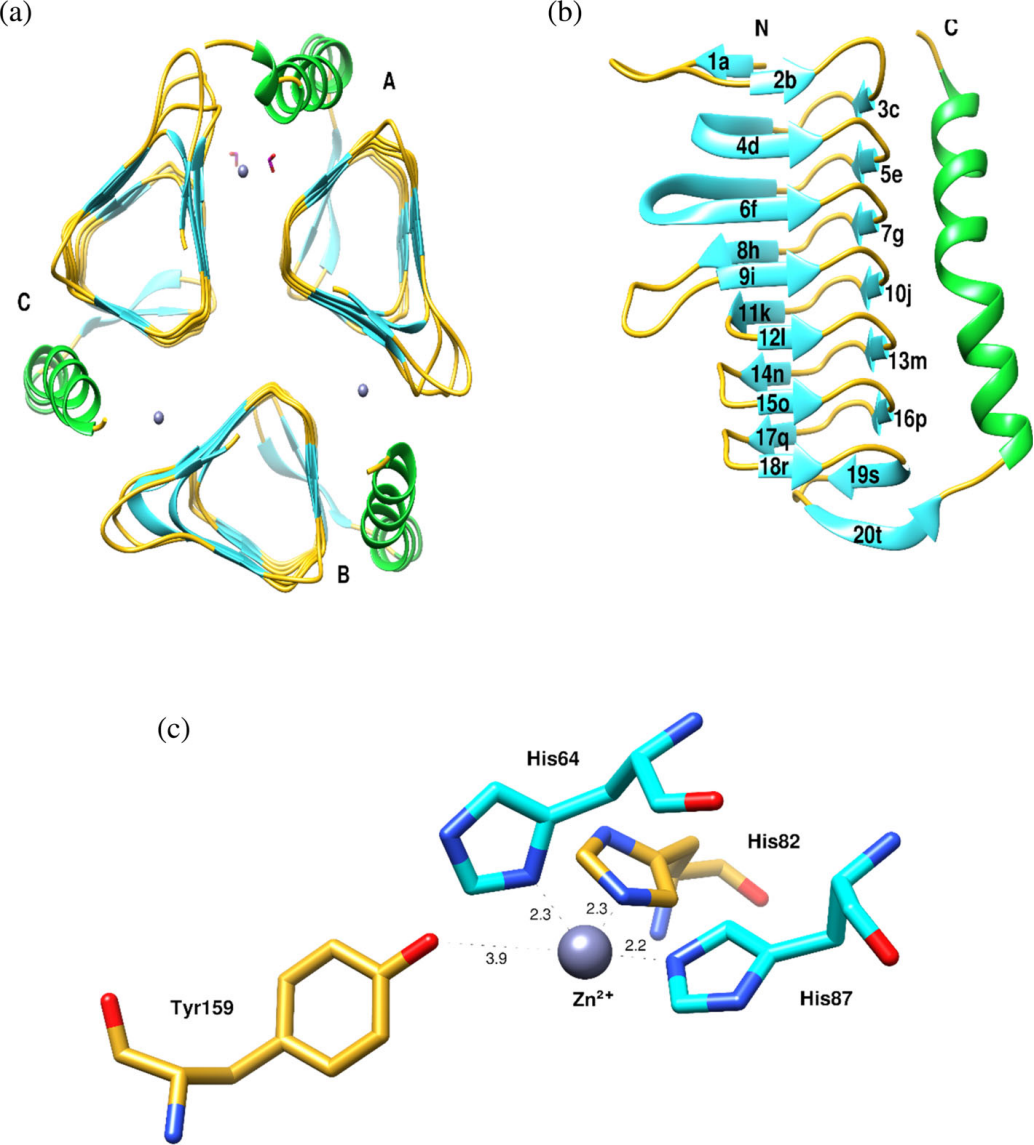
(a) Overall ribbon diagram of the trimeric γ*‐Ca*CA. Strands, coils and helices in each monomer are colored as cyan, green and goldenrod, respectively. The zinc ions are depicted as spheres in gray color. Formate ions (FMTs) are shown as sticks in purple color. (b) Ribbon diagram of γ*‐Ca*CA (chain B). Secondary structure elements were assigned by KSDSSP in Chimera (Pettersen et al., 2004). Strands are colored in cyan and the C‐terminal helix in green. (c) Close up view of the zinc‐binding site between monomers B and C. Distances are shown in Å units. Figures were created using Chimera.

γ‐*Ca*CA was crystallized with three molecules (A, B, C) in the asymmetric unit (Table 2 and Figure 3a). The trimer structure has a surface area of 19,270 Å^2^ and a buried surface area of 6830 Å^2^, whereas chain A has a surface area of 8627.3 Å^2^. The rmsd values in Cα positions observed between molecule pairs A‐B, B‐C, and C‐A are 0.134, 0.128, and 0.128 Å, respectively. The three molecules show subtle changes, mainly owing to the flexibility of the C‐terminal 6xHis‐tag. The overall architecture consists of seven rounds of parallel coil‐beta strands and an antiparallel beta strand (residues 139–143) and an alpha helix (Glu146–Tyr166) (Figure 3b). Each chain is shaped as a horizontal triangular cylinder and is connected to the neighboring chain by zinc‐mediated interactions at the interface. Twelve sodium cations and two formate anions (FMT1 and FMT2) are present in the structure. Both FMTs are at the interface of chain A and chain C (Figure 3a). FMT1 lies in the proximity of the zinc ion, whereas FMT2 is located beneath but away from FMT1 near the start of the α‐helix.

**TABLE 2:** X‐ray data collection and refinement statistics.

Data collection	γ*‐Ca*CA	γ*‐Ca*CAmut
Beamline	P13 (EMBL, Hamburg)	P13 (EMBL, Hamburg)
Wavelength (Å)	0.8266	1.0
Resolution range (Å)	49.25–1.11 (1.15‐1.11)	47.48–2.10 (2.17–2.10)
Space group	*P* 2_1_ 2_1_ 2_1_	*P* 2_1_ 2_1_ 2_1_
Unit cell		
*a, b, c* (Å)	57.02, 82.84, 98.51	81.69, 164.96, 165.11
*β* (°)	90	90
No. of unique reflections	184,092 (17,915)	130,473 (12,671)
Completeness (%)	99.9 (99.7)	99.9 (99.7)
Multiplicity	13.0 (12.7)	13.7 (13.8)
Mosaicity (°)	0.18	0.14
*R*_meas_	0.086 (4.016)	0.165 (3.053)
CC_1/2_	0.999 (0.416)	0.999 (0.427)
Mean (I/σ(I))	12.2 (0.8)	10.6 (1.0)
Wilson B‐factor (Å^2^)	14.5	49.9
*Refinement*		
No. of reflections used	183,904	130,317
*R*_cryst_/*R*_free_	0.166/0.188	0.20/0.24
No. of non‐H atoms (protein/ligand/solvent)	3,924/21/577	12,383/86/472
RMSD in bonds (Å)	0.005	0.007
RMSD in angles (°)	0.894	0.912
Average B‐factor (Å^2^)	21.3	53.6
Ramachandran favored/outliers (%)	97.4/0.0	94.9/1.1
Rotamer outliers (%)	0.0	2.4
Clashscore	5.5	6.0
PDB id	9QEV	9QEZ

The active site of each γ*‐Ca*CA chain is located at the interface of neighboring chains A‐B, B‐C and C‐A. There are three zinc ions, coordinated by His64, His82, His87 and Tyr159 (Figure 3c), at the active site. His64 and His87 belong to the same monomer whereas His82 belongs to the neighboring chain, resulting in a distorted trigonal bipyramidal structure for the Zn^2+^ coordination.

### 2.5 Structural comparison and the basis of low activity of γ‐ *Ca*CA

**FIGURE 4:**
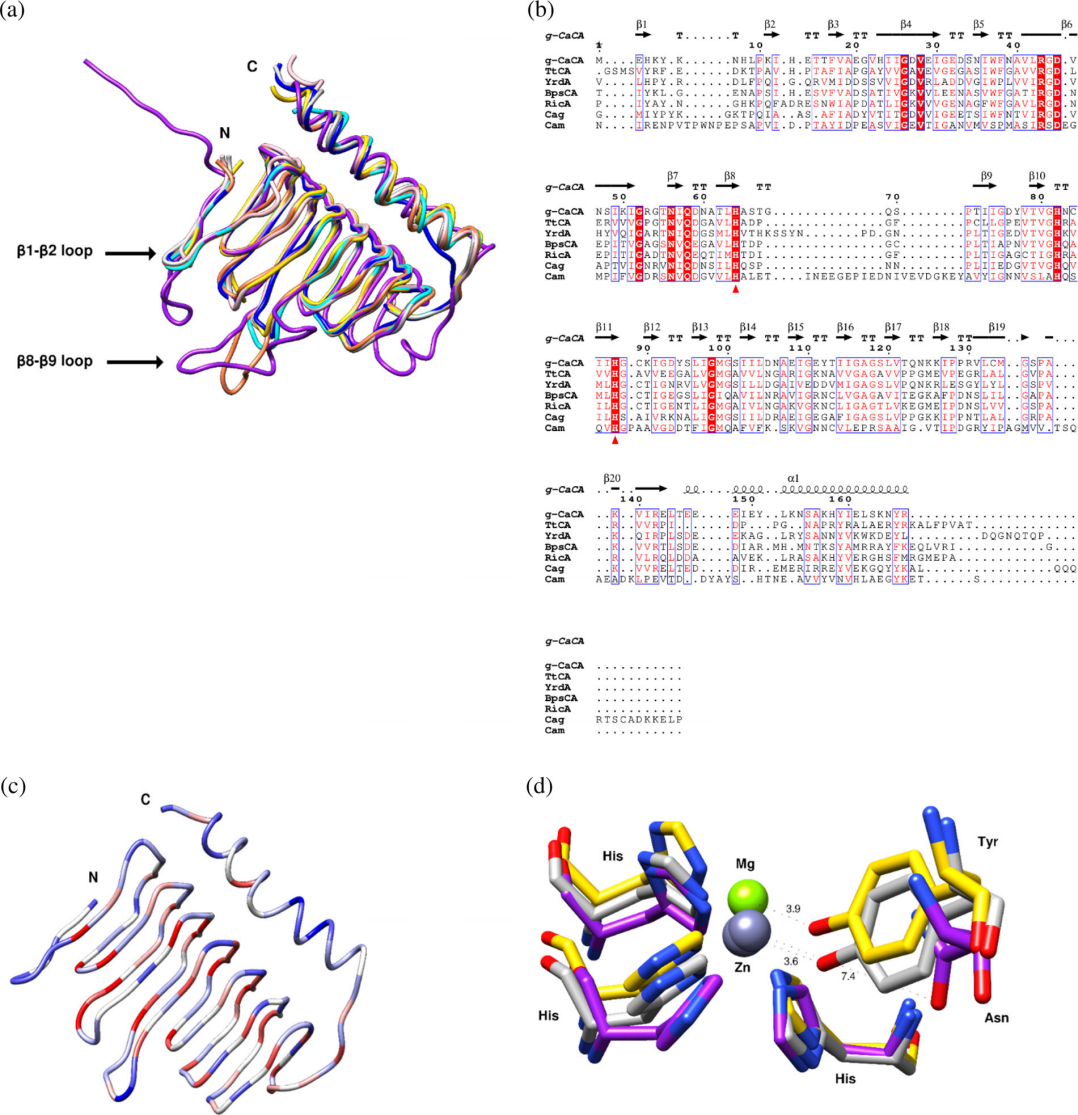
Structural comparisons and analysis of γ‐CAs. CAs from *Clostridioides difficile* (Cag; 4MFG), *Thermus thermophilus* HB8 (TtCA; 6IVE), *E. coli* (YrdA; 3TIO), *Brucella abortus* (RicA; 4N27) and *Methanosarcina thermophila* (Cam; 1THJ) were selected for comparative analysis. (a) Loop comparison among the PDB structures. γ‐*Ca*CA is colored as cyan and compared with TtCA (blue), YrdA (coral), *Bps*CA (gray), RicA (pink), Cag (gold) and Cam (purple). (b) Structure‐based sequence alignment. Conserved residues are highlighted in red columns. Conserved residues at the active site are marked by red triangle symbol underneath. This figure was created with ESPript (Robert & Gouet, 2014). (c) Sequence conservation in γ‐CAs. Red denotes 100% conservation and blue 0% conservation. The structure‐based alignment in (b) was used. (d) C‐terminal helix residues interacting with the metal ion. The residues that belong to *Bps*CA, Cag, and Cam are colored gray, gold and purple, respectively. This figure was made using Chimera.

PDBeFold and PDBePISA were employed to search for structures similar to γ*‐Ca*CA for structural comparison. CAs from *Clostridioides difficile* (Cag; PDB: 4MFG), *Thermus thermophilus* HB8 (*Tt*CA; PDB: 6IVE), *E. coli* (YrdA; PDB: 3TIO), *Brucella abortus* (RicA; PDB: 4N27) and *Methanosarcina thermophila* (Cam; PDB: 1THJ) were selected for comparative analysis. The selected γ‐CAs show very different levels of catalytic activities, spanning from inactive proteins such as *Tt*CA (Wang et al., 2019), YrdA (Park et al., 2012) and RicA (Herrou & Crosson, 2013) to active proteins such as Cam (Kisker et al., 1996), Cag (Sridharan et al., 2021), and *Bps*CA (Di Fiore et al., 2022). The observed RMSD values, ranging from 0.65 to 0.80 Å, indicate minor structural deviations and a high degree of overall structural conservation, as illustrated in Figure 4a. A structure‐based sequence alignment is shown in Figure 4b.

It is well established that CAs catalyze a two‐step ping‐pong reaction: (i) nucleophilic attack of metal bound OH^−^ on CO_2_ resulting in HCO_3_^−^ and H_2_O formation, and (ii) transfer of proton from metal bound H_2_O to the buffer. During this process, the metal ion functions as a Lewis acid reducing the p*K*_*a*_ of water molecule. The second step is the rate‐limiting step and is governed by a proton shuttle residue. Glu85 and Glu123 have been identified as proton shuttle residues in Cam and *Bps*CA, respectively (Di Fiore et al., 2022; Zimmerman et al., 2010). Proton shuttle residues in these CAs are found in close proximity to the active site and have been proposed to contribute to stability and activity (Mikulski & Silverman, 2010). In γ‐*Ca*CA, the key catalytic residues identified in Cam, Arg43, Asn56, and Gln58 (Kisker et al., 1996), are conserved. However, other functionally important residues in Cam, including Asn203, Glu63, and the putative proton shuttle residue Glu85 are absent in γ‐*Ca*CA.

Despite the overall structural conservation, differences were observed in β1‐β2 and β8‐β9 loop residues which were found to be less conserved (Figure 4b). The residues of the β8‐β9 loop are pointing towards the active site. Interestingly, Cam is the only active CA with a longer β1‐β2 and β8‐β9 loop among the compared CAs. Second to Cam is Cag (Sridharan et al., 2021) which is another active CA but lacks a long β8‐β9 loop. *Bps*CA, another active CA among the compared CAs, is characterized by short β1‐β2 and β8‐β9 loops. Comparison of the three active CAs (Cam, Cag, and *Bps*CA), indicates the absence of any established pattern or mechanism that may characterize a CA as active or inactive. Interestingly, YrdA (Figure 4b), despite being inactive, exhibits a longer β8‐β9 loop, further supporting the argument that a defined pattern for an ‘active γ‐CA’ is yet missing.

The potential role of the C‐terminal helix in the activity of some γ‐CAs was also examined. Structure‐based sequence alignment (Figure 4b) and conservation diagrams (Figure 4c) showed low sequence conservation in the C‐terminal helix. Tyr159 in γ‐*Ca*CA is notably highly conserved among the compared CAs, with the exception of Cam, where it is substituted by an Asn residue (Asn203) (Figure 4b,d). In γ*‐Ca*CA, Tyr159 points towards the active site, and the hydroxyl group is ~3.9 Å from the zinc ion. In Cam, Asn203 is located at 6.6 Å from the zinc ion. In *Bps*CA, which is characterized by moderate activity, Tyr159 is 3.6 Å from the zinc ion. In Cag, Tyr158 is 3.9 Å from the magnesium ion (Figure 4d). The analysis indicated that there is no established pattern of amino acid residues from the C‐terminal helix or a certain distance to the metal atom that may determine whether a CA is active or inactive.

### 2.6 Design and evaluation of an engineered γ*‐* *Ca*CAmut

#### 2.6.1 Design of the mutant enzyme γ‐CaCAmut

**FIGURE 5:**
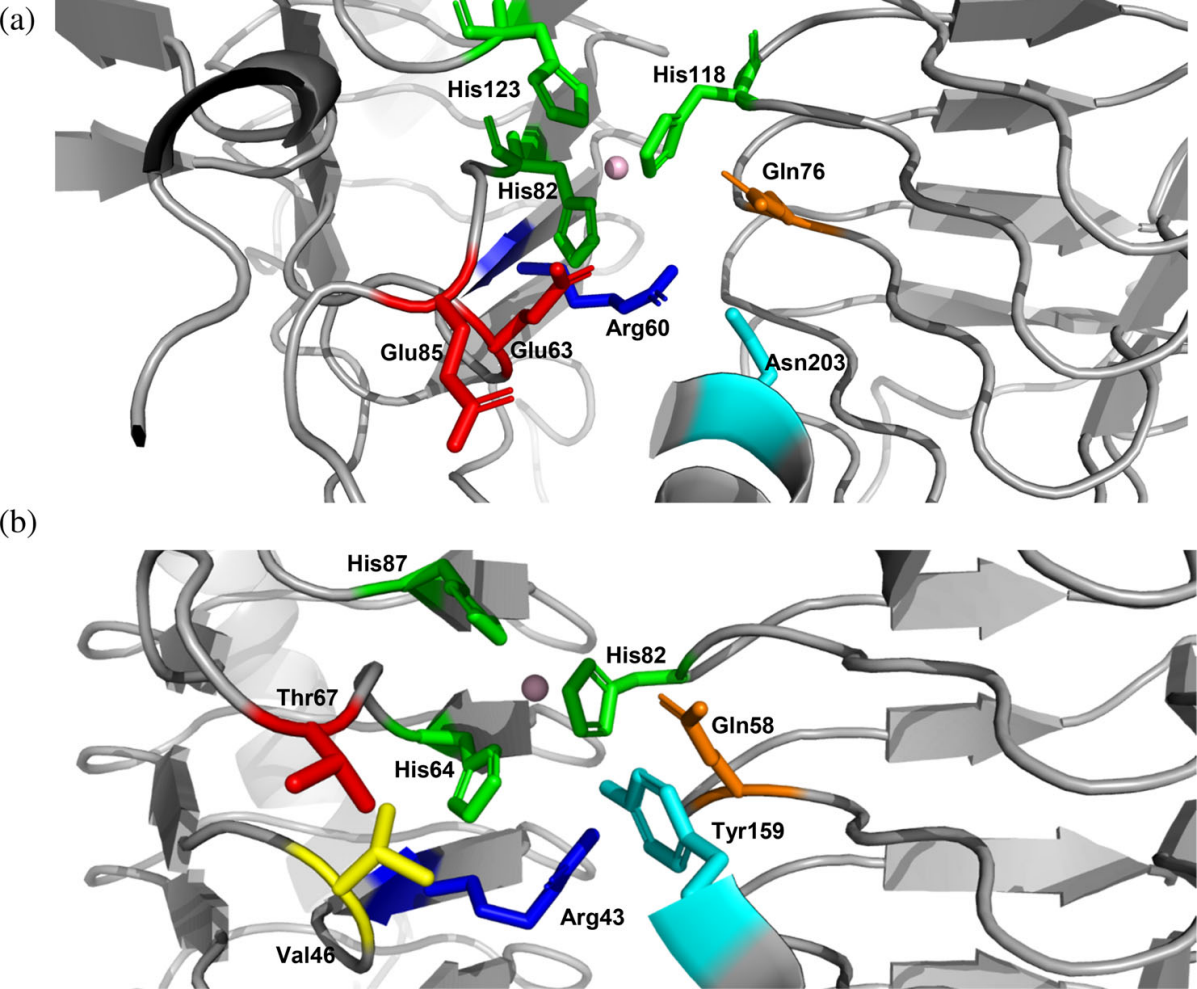
Comparison of selected active site residues in (a) Cam from *M. thermophila* (PDB ID:1THJ) (b) γ*‐Ca*CA. The bound zinc ions in the active sites are shown as spheres.

Sequence and structural analysis of the catalytically active Cam identified three conserved His residues (His82, His118, and His123) that are involved in metal binding and a number of other residues (Arg60, Glu63, Gln76, Glu85, Asn203) that contribute to catalysis and formation of the active site (Figure 5a and Table 3). A comparison between the active centers of γ‐*Ca*CA and the Cam homolog reveals that γ*‐Ca*CA lacks Glu63, Asn203and the essential acidic loop with the proton shuttle residue Glu85 (Figure 5b and Table 3). The residues Gln76, Asn203, and Glu63 in Cam, which play significant roles in positioning carbon dioxide in the active site, are replaced by Gln58, Tyr159, and Val46, respectively, in γ*‐Ca*CA. In Cam, Glu63 is essential for releasing the reaction product, which is in a hydrogen bond with bicarbonate and is also involved in the transfer of protons that are produced by the formation of hydroxide from the water attached to zinc. These discrepancies between the two enzymes prompted the question of whether a catalytic function can be engineered for γ*‐Ca*CA. Consequently, an engineered form of γ*‐Ca*CA was designed using the structural information from the homolog Cam enzyme.

**TABLE 3:** Comparison of essential amino acid residues in Cam with those in γ‐*Ca*CA for the design of the engineered enzyme γ*‐Ca*CAmut.

Enzymes	Residues							
Cam	Arg60	Glu63	Gln76	His82	Glu85	His118	His123	Asn203
γ*‐Ca*CA	Arg43	**Val46**	Gln58	His64	**Thr67**	His82	His87	**Tyr159**
γ*‐Ca*CAmut	Arg43	**Glu46**	Gln58	His64	**Glu67**	His89	His94	**Asn166**

*Note*: The residues that were targeted for mutagenesis in γ‐*Ca*CA and the mutated residues in γ*‐Ca*CAmut are depicted with bold letters.

Based on the analysis described above, our engineering strategy involved substituting Val46 and Tyr159 of γ‐*Ca*CA with Glu and Asn, respectively (Table 3). Furthermore, our strategy involved incorporating the acidic loop segment from the Cam sequence (Glu^85^‐Thr‐Ile‐Asn‐Glu‐Glu‐Gly‐Glu‐Pro^93^‐), which encompasses the proton shuttle residue Glu85. This modification consequently resulted in two additional amino acid substitutions, with Thr67 and Gly68 of γ‐*Ca*CA being replaced by Glu residues. To validate our structure‐based design, a mutant form of γ‐*Ca*CA was constructed (named γ‐*Ca*CAmut) and its kinetics and structural properties were investigated.

#### 2.6.2 Biochemical characterization of γ‐CaCAmut

**FIGURE 6:**
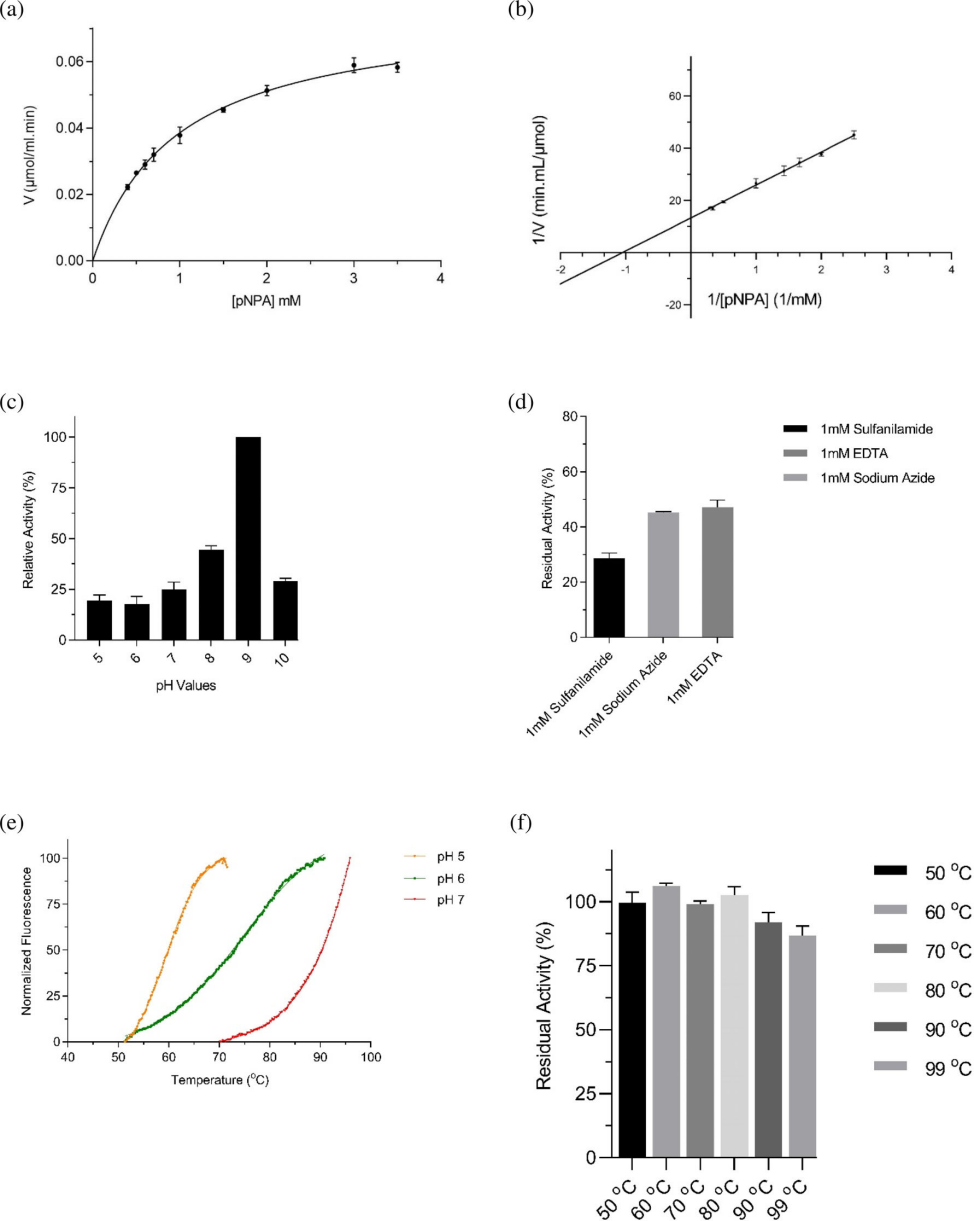
(a) Plot of the initial rates of p‐NPA hydrolysis by γ‐*Ca*CAmut at 25°C, with a Michaelis–Menten curve fitted to the data. (b) Lineweaver‐Burk plots of the hydrolysis of pNPA catalyzed by γ‐*Ca*CAmut at 25°C. (c) pH dependence at 25°C of the initial rates of pNPA hydrolysis catalyzed by γ‐*Ca*CAmut. (d) Inhibition of γ‐*Ca*CAmut by common CA inhibitors (sulfanilamide, sodium azide, EDTA). The activity of γ‐*Ca*CAmut was assayed using pNPA as substrate at 25°C. The concentration of the inhibitors was 1 mM. (e) DSF curves of γ‐*Ca*CAmut for the determination of melting temperature (*T*_*m*_) at pH 5.0, 6.0 and 7.0. Curves were fit with the Boltzmann sigmoidal model (solid lines). Goodness of fit (*R* ^2^) values for each pH are 0.99, 0.98 and 0.98, respectively. (f) Thermal inactivation profile of γ‐*Ca*CAmut at pH 8.0. The enzyme was incubated for 5 min at temperatures ranging from 50°C to 99°C, and residual esterase activity was measured using pNPA as substrate.

γ‐*Ca*CAmut was expressed and purified as described for the wild‐type enzyme (Figure S1b). The catalytic activity of purified γ‐*Ca*CAmut was assessed towards the two model reactions, similar to that performed for the wild‐type enzyme. The results demonstrated that γ‐*Ca*CAmut exhibits approximately twofold higher CO₂ hydration activity compared with the wild‐type enzyme (Figure S2). In addition, it displayed esterase activity using p‐NPA as substrate. The esterase activity of γ‐*Ca*CAmut obeyed normal Michaelis–Menten saturation kinetics (Figure 6a,b) with kinetic parameters *K*_*m*_ and *k*_cat_ 0.96 ± 0.05 mM and 4.46 min^−1^, respectively. The determined *K*_*m*_ values align well with those reported for other CAs, such as from the thermophilic bacterium *Sulfurihydrogenibium* and bovine, with values 2.8 and 3.4 mM, respectively (Capasso, de Luca, et al., 2012). However, the *k*_cat_ of γ‐*Ca*CAmut is significantly lower (approximately 10^3^–10^4^ times) compared to that of the enzymes from *Sulfurihydrogenibium* and bovine.

The pH dependence of esterase activity was also assessed (Figure 6c). The results showed an alkaline pH optimum similar to that found in other CAs, such as those from human erythrocytes (Verpoorte et al., 1967). Inhibition of γ‐*Ca*CAmut by three common diagnostic CA inhibitors (sulfanilamide, azide, and EDTA) was also evaluated (Figure 6d). Consistent with findings for other carbonic anhydrases (Supuran, 2016b), the observed inhibition further supports the conclusion that γ‐*Ca*CAmut exhibits characteristics typical of this enzyme family.

The structural stability of γ‐*Ca*CAmut was further evaluated using DSF in different pH values, similar to those conducted for the wild‐type enzyme (Figure 2). The melting curves are shown in Figure 6e and the measured *T*_*m*_ values are listed in Table 1. The results demonstrated that γ‐*Ca*CAmut exhibited significantly enhanced thermostability, with its *T*_*m*_ value at pH 7.0 increasing by approximately 18°C compared to the wild‐type enzyme. Interestingly, the melting curve at pH 8.0 in the DSF experiment failed to reach maximum fluorescence before the complete denaturation of γ‐*Ca*CAmut. This is because of the limitations of the PCR instrument (maximum temperature 99°C). Therefore, as an alternative approach for assessing the thermostability, we employed thermal inactivation studies, exploiting the esterase activity of γ‐*Ca*CAmut. Figure 6f illustrates the thermal inactivation profile of γ‐*Ca*CAmut at pH 8.0 over the temperature range of 50–99°C. The results indicate that the enzyme remained catalytically active throughout this temperature range, and retained more than 80% (at 99°C) of its initial activity, indicating exceptional thermostability.

### 2.7 Crystal structure analysis of γ‐ *Ca*CAmut

**FIGURE 7:**
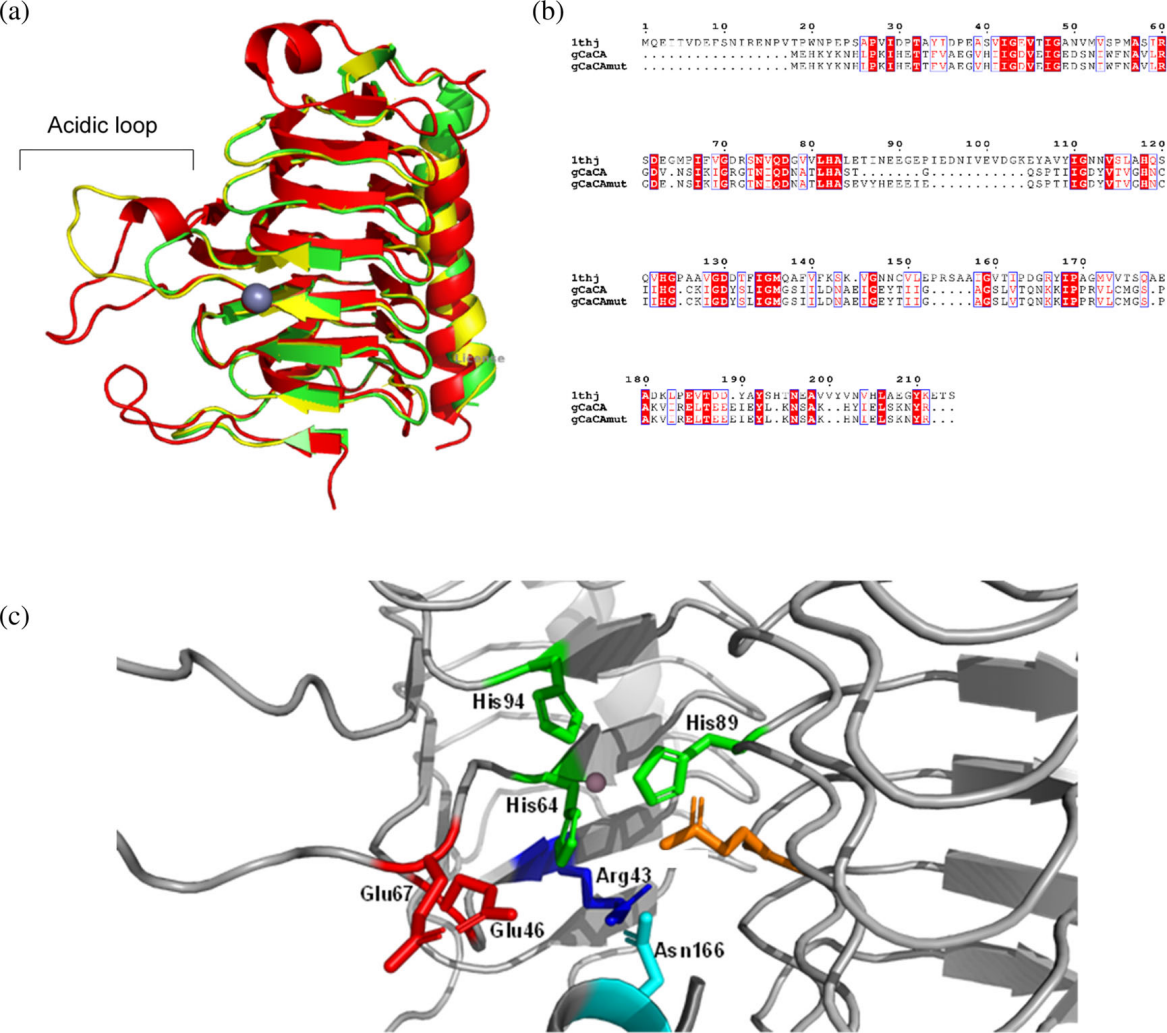
(a) Structural overlay of the γ*‐Ca*CA monomer (green), the γ*‐Ca*CAmut (yellow) and γ‐CA from Cam (red) (PDB ID: 1THJ). The position of the acidic loop is labeled. (b) Sequence alignments of γ‐CA from Cam (PDB ID: 1THJ), γ‐*Ca*CA and γ‐*Ca*CAmut. Alignment was performed using ClustalO and displayed using ESPript 3.0. Conserved areas are shaded in red columns. (c) Selected active site residues in γ‐*Ca*CAmut. The bound zinc ion in the active site is shown as sphere.

The newly adapted esterase activity and the dramatic increase in *T*_*m*_ of the γ‐*Ca*CAmut prompted us to investigate its 3D structure using X‐ray crystallography (Figure 7). The mutant enzyme was crystallized, and its structure was determined at 2.1 Å resolution. The asymmetric unit was found to contain nine molecules of γ‐*Ca*CAmut arranged in three homotrimers, each of which is similar to the wild‐type typical homotrimer. Structural superposition of the two structures revealed an rmsd of 0.393 Å, suggesting subtle changes. Key differences were observed in the β8‐β9 loop after the insertion. The incorporation of extra residues made the loop highly flexible and difficult to model owing to poor electron density. Zinc was also found at the active site of γ‐*Ca*CAmut and the catalytic residues, including His and Tyr, were found in similar positions in both the wild‐type and mutant structures. The orientation of the mutated residues Asn166 and Glu67 towards the active site suggests their possible interactions with zinc and role in the catalytic efficiency and stability of the active site.

### 2.8 Structural basis of γ‐ *Ca*CAmut esterase activity

**FIGURE 8:**
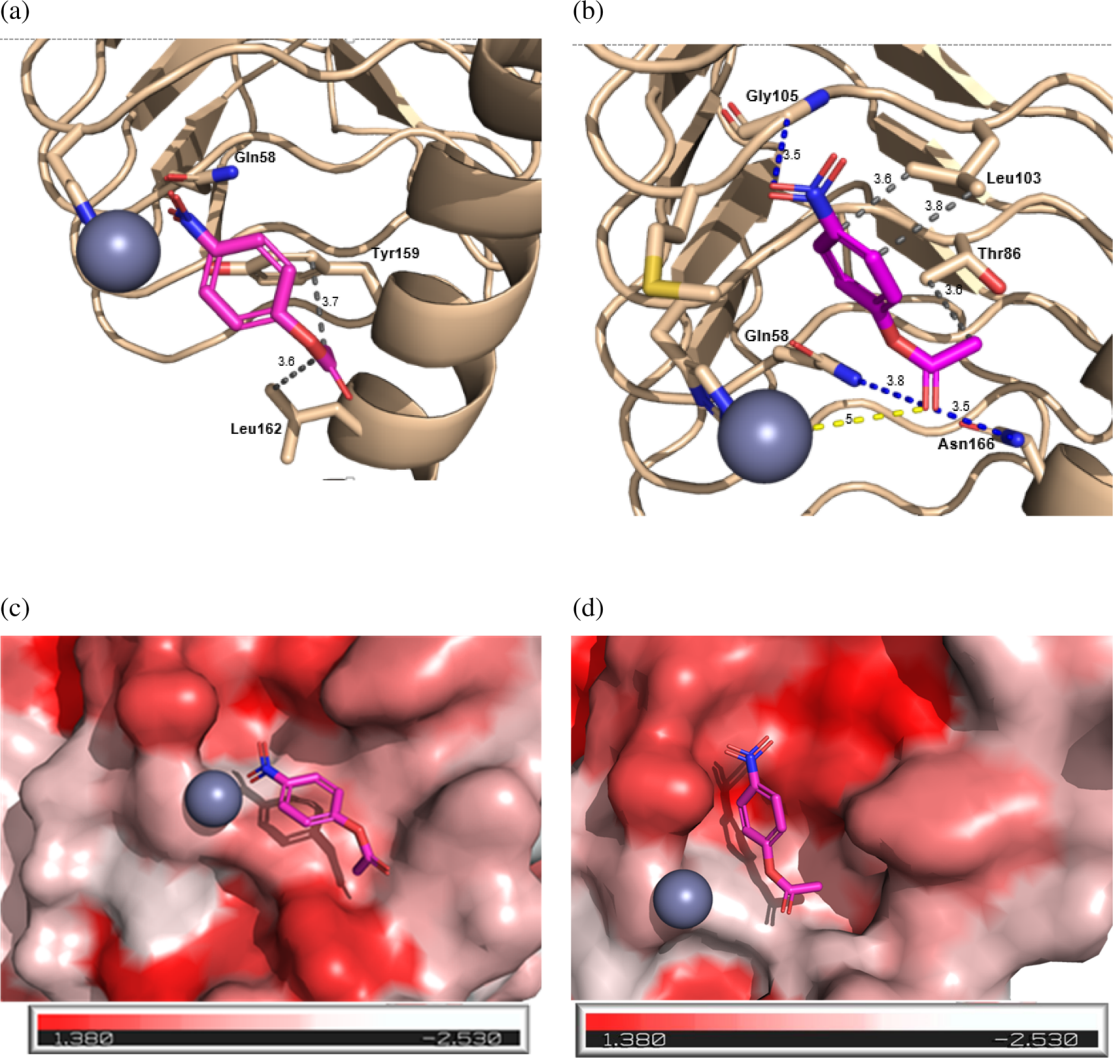
(a, b) The most favorable predicted mode of γ‐*Ca*CA (a) and γ‐*Ca*CAmut (b) interaction with pNPA. Blue and gray dashed lines represent H‐bond and van der Waals interactions, respectively. pNPA is shown as a stick representation and colored according to the atom type. The figures were created using the SwissDock server. (c, d) Hydrophobicity potential of γ‐*Ca*CA (c) and γ‐*Ca*CAmut (d). Red and white shades represent hydrophobic and hydrophilic regions, respectively, based on the Eisenberg's scale. (e) The predicted mode of interaction of γ‐*Ca*CA with the pNPA. Side chains of residues in contact with the ligand are shown with dashed lines. (f) The proposed interaction mechanism between γ‐*Ca*CAmut and pNPA. Dashed lines indicate the side chains of residues that interact with the ligand. pNPA is shown as a stick representation and colored purple. The figures were created using the PyMOL program.

Building on the high‐resolution structures determined in this study, molecular docking analyses were performed to examine the interactions of p‐NPA with γ‐*Ca*CA and γ‐*Ca*CAmut, with the objective of elucidating the experimentally observed differences in catalytic activity. Figure 8 illustrates the most probable binding modes of p‐NPA to both γ‐*Ca*CA and γ‐*Ca*CAmut. p‐NPA binds to two enzymes with two distinct binding orientations: a productive (γ‐*Ca*CAmut) and a non‐productive (γ‐*Ca*CA). In the case of γ‐*Ca*CA, p‐NPA binds in the active site with its susceptible ester bond oriented opposite to the Zn^2+^ (Figure 8a). On the other hand, p‐NPA binds in the active site of γ‐*Ca*CAmut (Figure 8b), with its ester carbonyl oxygen close to the catalytic Zn^2+^. The main driving force that fixes the substrate to two distinct orientations appears to be variations in the hydrophobicity of the two binding sites (Figure 8c,d). Analysis of the γ‐*Ca*CAmut structure showed that Leu103 contributes to the stabilization of the aromatic ring of p‐NPA in the active site by creating two hydrophobic contacts with the aromatic group (Figure 8b). Furthermore, Gly105 makes an H‐bond with the nitro group of the substrate. These interactions seem to contribute towards fixing the orientation of the ester bond, allowing Gln58 and the newly mutated residues Asn166 to form hydrogen bonds with the carbonyl oxygen of p‐NPA (Figure 8b). In addition, the methyl group of Thr86 further contributes to the orientation of the ester group by forming a van der Waals contact with the methyl group of acetate. In this orientation and assuming that the mutant enzyme retains the conserved catalytic mechanism of CAs, a water molecule coordinated to the Zn^2+^ ion can be positioned to act as a nucleophile, initiating the attack on the carbonyl carbon of the ester substrate. All these interactions are absent in γ‐*Ca*CA (Figure 8a), resulting in unproductive binding of p‐NPA and the absence of catalytic activity.

### 2.9 Structural basis of γ‐ *Ca*CAmut hyperthermostability

The enhanced thermostability of γ‐*Ca*CAmut can be attributed to multiple factors, including the presence of additional residues in the acidic loop, improved electrostatic interactions, increased solubility, or oligomerization mediated by various interfacial interactions among the contributing monomers (Fraser et al., 2016).

Oligomerization has been implicated in other enzymes as a potential factor of thermostability (Fraser et al., 2016). To test this hypothesis, analysis of the oligomerization stage of γ‐*Ca*CAmut at pH 8.0 was accomplished using mass photometry. The results demonstrated (see Figure S3) that only a trimeric structure was present in solution. It is therefore possible that the oligomerization observed in the crystals is a crystallization artifact. However, the concentration used in mass photometry measurements is low (50 nM) and the protein may still be able to oligomerize at high concentrations, such as those found in the crystals.

To investigate whether the solubility factor contributes to the enhanced stability of γ‐*Ca*CAmut, we compared the predicted pI and scaled solubility value using the Protein‐Sol *in silico* tool (Hebditch et al., 2017). We found that for the wild‐type enzyme the pI and the scaled solubility value were 6.58 and 0.671, respectively. On the other hand, for the γ‐*Ca*CAmut, the predicted pI and solubility were 6.08 and 0.862, respectively, suggesting that the mutant enzyme displays significantly higher solubility, a crucial factor that affects protein stability (Qing et al., 2022).

**FIGURE 9:**
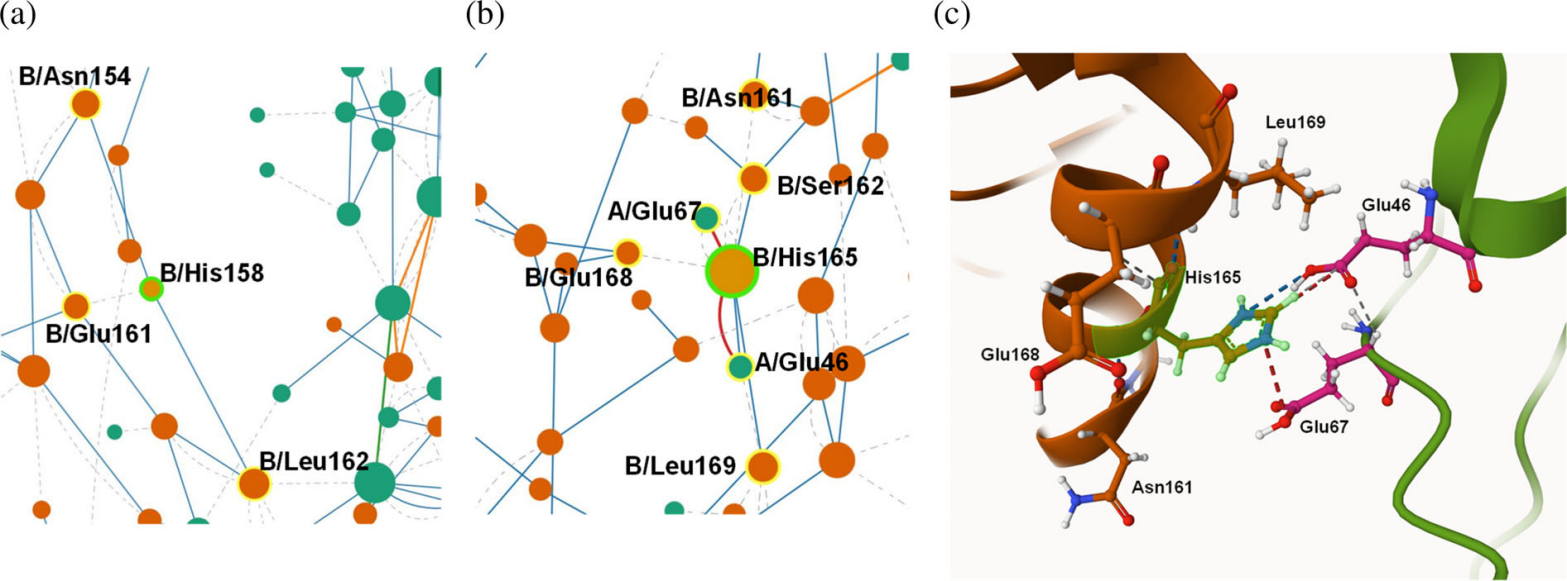
(a) Residue interaction network around B/His158 of γ‐*Ca*CA. (b) Residue interaction network around B/His165 of γ‐*Ca*CAmut. (c) Interactions formed between B/His165 with A/Glu46, A/Glu67 and other nearby residues. Pictures were created using the RING 4.0 web server (del Conte et al., 2024). Blue, red, and gray dashed lines represent H‐bond, ionic and van der Waals interactions, respectively.

Furthermore, residue interaction network using the RING 4.0 program (del Conte et al., 2024) was used in order to identify important interactions in γ‐*Ca*CAmut that may contribute to its enhanced thermostability. Numerous inter‐chain interactions were identified. In the case of γ‐*Ca*CA a total of 19 H‐bonds, three ionic and 24 van der Waals interactions are observed. Interestingly, a larger number of inter‐chain interactions were found in γ‐*Ca*CAmut: 22 H‐bonds, nine ionic and 46 van der Waals inter‐chains. Furthermore, the two engineered amino acids in the γ‐*Ca*CAmut structure (Glu46 and Glu67) seem to contribute substantially to thermostability, since they interact with His165 of the neighboring subunit, forming two ionic bonds, one H‐bond, and two van der Waals interactions (Figure 9). It is highly plausible that these newly formed electrostatic interactions and the higher solubility of the mutant enzyme may be key factors that contribute significantly to trimer stabilization, enhancing the enzyme's overall thermostability.

## 3 CONCLUSIONS

In this study, a γ‐CA with 95% sequence identity to *Caloramator australicus* γ‐CA was identified in a geothermal spring metagenomic soil sample and characterized. The enzyme displayed marginally low CO₂ hydration activity and no measurable esterase activity. The crystal structure of the native enzyme was determined at 1.11 Å resolution, the highest resolution reported to date for any γ‐CA, allowing detailed analysis of its active site architecture. Structural inspection revealed the absence of key catalytic residues and elements of the C‐terminal helix, explaining the observed inactivity. To probe the functional consequences of these missing elements, a rational design approach was employed to introduce the absent catalytic residues. The engineered variant displayed slight increased CO₂ hydration activity, restored esterase activity towards p‐NPA and a substantial increase in thermostability compared to the wild‐type enzyme. Residues critical for CO₂ hydratase activity (e.g., Glu63, Asn203) were introduced via site‐directed mutagenesis. The resulting mutant enzyme exhibited increased, yet still overall low, hydratase activity, highlighting the complex structural requirements underlying this catalytic function. High‐resolution structural characterization of the mutant enzyme provided insights into the conformational adaptations associated with its acquired catalytic function and enhanced stability. Collectively, these findings expand our understanding of structure–function relationships in γ‐CAs and highlight the potential of rational protein engineering to rescue or repurpose inactive enzyme scaffolds. The engineered variant, with its unique combination of hyperthermostability, metal‐binding capacity, and esterase activity, represents a valuable template for future biotechnological and industrial applications. Its stability and catalytic versatility indicate strong potential for biocatalytic applications under extreme conditions (Burkhardt et al., 2023).

## 4 MATERIALS

The expression vector pETite C‐His Kan was contained in the Expresso™ T7 Cloning and Expression System (Lucigen, Middleton, WI). KAPA High Fidelity DNA polymerase was purchased from KAPA Biosystems (Pty, Cape Town, South Africa). Cloning was accomplished using the In‐Fusion® HD Cloning Kit (Takara Bio USA Inc., Mountain View, CA). The p‐nitrophenyl acetate (p‐NPA), kanamycin, and other analytical grade chemicals and salts were obtained from Sigma‐Aldrich (St. Louis, MO).

## 5 METHODS

### 5.1 Sample collection and DNA extraction

Wet soil samples from a thermal spring at Lisvori, Greece (39^o^06′03.5″ N, 26^o^12′04.3″ E) were collected aseptically in sterile tubes in May 2019. The in situ temperature was 70°C. Following storage at 4°C for a short period of time, extraction of total environmental DNA (eDNA) was carried out with a DNeasy® PowerMax® Soil kit (QIAGEN, Germany), using 10 g of soil sample, according to the manufacturer's instructions. The eDNA was concentrated by precipitation with NaCl/cold ethanol, and the resulting pellet was washed with an ethanol solution (70% v/v). The quality of the purified eDNA was evaluated by agarose gel electrophoresis and quantified using a NanoDrop® ND‐1000 spectrophotometer (Thermo Fisher Scientific Inc., MA). The final yield was approximately 10.1 ± 0.5 ng‧μL^−1^ (Average ± SD).

### 5.2 Amplification of 16S rRNA for high throughput sequencing

The hypervariable region V3‐V4 of the 16S rRNA gene was targeted for amplification, producing a ~460 bp fragment. The specific primer set comprised 341F (5′‐CCTACGGGNGGCWGCAG‐3′) (Herlemann et al., 2011; Klindworth et al., 2013) and 805 Rmod (5′‐GACTACNVGGGTWTCTAATCC‐3′) (Apprill et al., 2015). Each oligonucleotide bore overhanging Illumina adapters, and the total eDNA was used as a template for the gene region of interest. The PCR amplification mixture, reaction conditions, 16S Metagenomic Sequencing Library Preparation (Illumina), and sequencing on an Illumina®MiSeq (PE300) platform were performed as described by Salmaso et al. (2018) (Salmaso et al., 2018). The produced amplified sequences were designated according to sample‐specific barcodes and saved as FASTQ‐formatted files. Sequencing data were deposited in the National Center for Biotechnology Information (NCBI) database and can be accessed via BioProject ID PRJNA997949.

### 5.3 Bioinformatics analysis

Raw data were obtained as FASTQ files, and their processing was accomplished using the QIIME 2™ microbiome analysis package, version 2019.4 (Bolyen et al., 2019). The analysis followed a series of steps as previously described by Pantiora et al. (Pantiora et al., 2024). Briefly, paired‐end reads belonging to the sample were subjected to quality control using DADA2 (Callahan et al., 2016) in which forward and reverse sequencing primers, low‐quality regions of the sequences, and chimeric products were subtracted. Subsequently, the remaining high‐quality sequences were merged into a single archive. The classification of the true non‐chimeric sequences (Amplicon Sequence Variants, ASVs) was carried out with SILVA 132 as a reference sequence alignment with majority‐consensus taxonomy (seven levels) clustered at 99% sequence similarity (Quast et al., 2013; Yilmaz et al., 2014). The Naïve Bayes algorithm was applied for the training of the classifier based on the multiple alignment of the SILVA 132 database (Bokulich et al., 2018; Pedregosa et al., 2012). The last steps of the analysis included the exception of the sequences pertaining to Archaea, chloroplasts, and mitochondria. Having assessed the sequencing depth, the number of reads was rarefied to 21,793 sequences, leading to a total of 214 classified ASVs (Weiss et al., 2017).

Sequences homologous to γ‐*Ca*CA were sought in the NCBI using BLAST (Altschul et al., 1990). The resulting sequence set was aligned with Clustal Omega (Thompson et al., 1994). ESPript (Robert & Gouet, 2014) was used for alignment visualization and manipulation.

### 5.4 Cloning and PCR amplification of γ‐ *Ca*CA like sequence

The complete gene of γ‐*Ca*CA was amplified by PCR from eDNA using the KAPA HiFi PCR Kit®. PCRs were performed using the primers, CaAF 5′‐GAA GGA GAT ATA CAT ATG GAG CAT AAA TAT AAA AAT C‐3′ and CaAR 5′‐GTG ATG GTG GTG ATG ATG CCT ATA ATT TTT AGA TAG TTC‐3′. The expression vector pETite was used for directional cloning of the amplified gene following the procedure of the In‐Fusion® HD Cloning Kit. The pETite plasmid contains the T7 promoter and a C‐terminal hexastidine (6His) tag. In order to amplify the full‐length ORF from eDNA, the PCR reaction was carried out in a total volume of 15 μL containing: 10 μM of each primer, 50 ng template genomic DNA, 10 mM dNTPs, 5 μL 5× KAPA HiFi Buffer, and 0.5 unit of KAPA HiFi DNA polymerase (KAPA Biosystems, USA). The PCR procedure consisted of 30 cycles of 20 s at 98°C, 15 s at 48°C, and 15 s at 72°C. A final extension time at 72°C for 10 min was performed after the 30th cycle. *Escherichia coli* DH5a competent cells were used as the host for gene cloning. Positive transformants were selected on Luria–Bertani (LB) plates containing kanamycin (30 μg/mL). Recombinant cells were screened by PCR‐colony using the T7F and CaAR primer pair. Positive clones were confirmed by direct DNA sequencing. The sequence of the γ‐*Ca*CA was deposited to GenBank database (accession number PV429983).

### 5.5 Construction of the γ‐ *Ca*CA mutant enzyme (γ‐ *Ca*CAmut)

The mutant γ‐*Ca*CA gene was obtained as a synthetic construct from Genscript Biotech Corp (New Jersey, United States) and cloned into a pETite expression vector as described for the wild‐type enzyme. CaAF and CaAR oligonucleotides were used as primers for the PCR reaction (see above). The mutated gene was confirmed by DNA sequencing.

### 5.6 Heterologous expression and purification of γ‐ *Ca*CA and γ‐ *Ca*CAmut

*E. coli* Rosetta™ 2(DE3)pLysS competent cells were transformed with the recombinant plasmids and cultured overnight at 37°C in LB medium supplemented with 30 μg/mL kanamycin. Starter cultures (5 mL) were then used to inoculate 500 mL LB containing kanamycin (30 μg/mL). Cultures were grown at 37°C and 180 rpm until the OD_600_ reached 0.5–0.6. Subsequently, ZnSO_4_ was added at a final concentration of 0.5 mM and the expression of the cloned gene was induced by the addition of 0.5 mM isopropyl 1‐thio‐β‐galactopyranoside (IPTG). Cultures were incubated at 37°C and 180 rpm for a further 4 h. The cells were then harvested using centrifugation (10,000×*g*, 4°C) for 10 min and stored at −20°C. The cell pellet (approximately 1 g) was resuspended in potassium phosphate buffer (50 mM, pH 8.0) containing sodium chloride (0.3 M), sonicated, and centrifuged (10,000×*g* for 10 min). Crude cell‐free extract was loaded onto a column of Ni‐IDA‐Sepharose (1 mL), which was previously equilibrated with potassium phosphate buffer (50 mM, pH 8.0) containing sodium chloride (0.3 M) and imidazole (10 mM). Bound γ*‐Ca*CA (or γ‐*Ca*CAmut) was eluted stepwise with equilibration buffer containing different concentrations of imidazole (20–300 mM). The collected fractions (2 mL) were collected and assayed for CA activity and protein (Kielkopf et al., 2020). The typical yield from 1 g cell pellet was about 30 mg of purified γ*‐Ca*CA or γ‐*Ca*CAmut. Protein purity was assessed using SDS‐PAGE (Laemmli, 1970). Before use, the purified enzyme fractions were dialyzed against Tris–HCl buffer (20 mM, pH 8.3) and stored at 4°C.

### 5.7 Assay of enzyme activity and kinetics analysis

The CO_2_ hydration activity of γ*‐Ca*CA or γ‐*Ca*CAmut was assayed by monitoring the pH variation due to the conversion of CO_2_ to bicarbonate, as previously described (Capasso, Luca, et al., 2012). Phenol red was used as the pH indicator and the assay was performed at 4°C using CO_2_‐saturated water as substrate. The assay buffer consisted of 20 mM Tris–HCl buffer (pH 8.3, containing 0.2 mM phenol red). An appropriate amount of enzyme solution was added to the test tube and an equivalent volume of buffer was added to the control tube. An appropriate amount of CO_2_‐saturated water was added and the time required for the solution to change from red to yellow (557 nm) was recorded. CO_2_ hydration activity was expressed in Wilbur‐Anderson (WA) units. One Wilbur‐Anderson unit (WAU) of CA activity is defined from the equation (T_0_ − T)/T, where T_0_, is the time (s) for the spontaneous (uncatalyzed) reaction, and T is the time (s) for the enzymatic reaction, required for the pH to drop from 8.3 to 6.3 in a control buffer and in the presence of enzyme, respectively. Stopped‐flow kinetic measurements (SFA20 model) were performed under ice‐cold conditions (0°C) using a final enzyme concentration of 19.1 μg/mL, as well as in the absence of enzyme (blank control). Four independent experimental repetitions were conducted, each consisting of three technical replicates. The reaction progress was monitored by measuring the decrease in pH from 8.3 to 6.3, using phenol red as a pH indicator.

The hydrolysis of p‐nitrophenyl acetate (p‐NPA) catalyzed by γ*‐Ca*CAmut was followed spectrophotometrically, as previously described (Jo et al., 2014), by monitoring the increase in absorbance for 180 s at 348 nm due to the formation of p‐nitrophenol at 25°C. The extinction coefficient ε = 5000 L × mol^−1^ × cm^−1^ for p‐nitrophenol was used. The reaction mixtures contained freshly prepared 3 mM p‐NPA (30 mM stock solution in acetonitrile) and 40 mM potassium phosphate (pH 7–8). The reaction was initiated by the addition of the enzyme. The rates of spontaneous hydrolysis were subtracted from the enzymatic rates. Steady‐state kinetics analysis of p‐NPA hydrolysis, catalyzed by γ‐*Ca*CAmut was carried out in 0.1 M KH_2_PO_4_, pH 8.0, at 25°C using different p‐NPA concentrations (0.4–3.5 mM).

### 5.8 Protein determination

Protein concentration was determined at 25°C by the method of Bradford (Kielkopf et al., 2020) using bovine serum albumin (fraction V) as the standard.

### 5.9 Electrophoresis

SDS polyacrylamide gel electrophoresis was performed according to the method of Laemmli (Laemmli, 1970) on a vertical slab gel containing 12.5% (w/v) polyacrylamide (running gel) and 2.5% (w/v) stacking gel. The protein bands were stained with Coomassie Brilliant Blue R‐250.

### 5.10 Thermal stability

The thermal stability of γ*‐Ca*CA and γ*‐Ca*CAmut was evaluated using differential scanning fluorimetry (DSF) on an Applied Biosystems® real‐time PCR StepOne™ instrument (Applied Biosystems, Waltham, MA, USA), according to (Huynh & Partch, 2015) (Huynh & Partch, 2015). Fluorescence was monitored between 15 and 99°C at a rate of 1°C/min. Melting temperatures (*T*_*m*_) were estimated using the Protein Thermal Shift Dye Kit™. *T*_*m*_ is defined as the temperature where both the folded and unfolded states are equally populated at equilibrium and was calculated by nonlinear fitting of the Boltzmann equation to the normalized fluorescence data. *T*_*m*_ values were measured in different buffer systems: 50 mM sodium acetate/NaOH, pH 5.5; 50 mM MES/NaOH, pH 6.0; 50 mM Tris/HCl, pH 7.0 and 8.0; 50 mM Glycine/NaOH, pH 9.0. The thermostability of γ‐*Ca*CAmut at pH 8.0 was assessed by measuring its residual esterase activity using pNPA as substrate, following heat treatment for 5 min at various temperatures ranging from 50°C to 99°C. Temperature control during incubation was maintained using a PCR thermocycler to ensure precise thermal conditions. To minimize evaporative losses during heating, an oil overlay was applied above the buffered enzyme solution. After heat treatment, the precipitated proteins (if formed) were removed by centrifugation and the supernatant was used for activity measurements. Residual activities were calculated as a percentage, relative to the activity of a non‐heated sample stored at 4°C.

### 5.11 Mass photometry

Mass photometry measurements were performed on a Refeyn TwoMP‐auto mass photometer. The protein was diluted to a final concentration of 50 nM in buffer Tris–HCl 10 mM (pH 8.0), NaCl 100 mM. The calibrant standards were bovine serum albumin and *Streptomyces rubiginosus* glucose isomerase. Both calibrants were diluted to 20 nM in the same buffer as the sample.

### 5.12 Structural determination by X‐ray crystallography

#### 5.12.1 Protein crystallization

The purified wild‐type protein was concentrated to 14 mg/mL in buffer HEPES 10 mM, NaCl 150 mM, and NaN_3_ 0.002 w/v, pH 7.0, prior to crystallization. Crystals suitable for X‐ray data collection were obtained with the hanging‐drop vapor diffusion method using a reservoir solution of 20% PEG 4 K and 0.2 M sodium formate. Single rod‐like crystals were observed after a few days. The mutant was concentrated to 10 mg/mL in the same buffer as that used for the native enzyme. Crystals were grown using 0.2 M magnesium chloride hexahydrate, 0.1 M HEPES (pH 7.5), and 30% v/v PEG 400. In both cases, the drops were prepared by mixing 2 μL of protein solution with 2 μL of reservoir solution and equilibrated at 16°C against 0.8 mL of reservoir solution.

#### 5.12.2 Data collection

Data to 1.11 Å resolution were collected remotely from a single γ*‐Ca*CA crystal at cryogenic temperature (100 K) on beamline P13 at Hamburg PETRA III synchrotron using an X‐ray wavelength of 0.8266 Å. Data processing revealed that the crystal belonged to the *P*2_1_2_1_2_1_ space group. Assuming three molecules in the asymmetric unit (a.u.), the Matthews coefficient was 2.04 D/Å^3^ (probability 1.0) corresponding to a solvent content of ~39% (Matthews, 1968). Data for the mutant (γ*‐Ca*CAmut) were collected remotely at 100 K with X‐rays at 1.000 Å wavelength (beamline P13). No cryoprotectant was added because of the high concentration of PEG 400 during crystallization. Assuming nine molecules in the a.u. for a primitive orthorhombic lattice (solvent content of ~60%), the Matthews coefficient was 3.1 D/Å^3^ (probability 0.99) (Matthews, 1968).

#### 5.12.3 Structure determination and refinement

Molecular replacement (MR) using Phaser (McCoy et al., 2007) as implemented in Phenix 1.20.1 (Adams et al., 2010) was applied to obtain the initial phases. The carbonic anhydrase from *Clostridioides difficile* (PDB ID: 4MFG; sequence identity: 57.7%) was used as a template for model creation. The sidechains were pruned with Sculptor (Bunkóczi & Read, 2011) to produce a suitable search model for MR. A unique initial solution was obtained with a TFZ score of 18.4. The obtained solution was iteratively refined using simulated annealing in PHENIX and maximum likelihood as the energy target. Coot (Emsley & Cowtan, 2004) was used to visualize and rebuild the structure to improve refinement statistics. *R*_free_ was monitored during refinement iterations along with other quality parameters such as Ramachandran plot and difference map peaks. The refined crystal structure of the wild‐type enzyme was used for molecular replacement in Phaser to solve the mutant structure. Refinement was performed using Phenix 1.20.1 as above. Data collection and final refinement statistics for both the wild‐type and mutant are shown in Table 2. The atomic coordinates and the structure factors for the wild‐type and the mutant enzyme have been deposited to the Protein Data Bank under the accession code 9QEV and 9QEZ, respectively.

#### 5.12.4 Structure validation and analysis

The final structures were validated with MOLPROBITY (Williams et al., 2018) and tools available in PHENIX (Adams et al., 2010) and COOT (Emsley & Cowtan, 2004). Structural correlations between γ*‐Ca*CA and deposited PDB structures were carried out using PDBeFold (Krissinel & Henrick, 2004). UCSF Chimera 1.13.1 (Pettersen et al., 2004) was utilized for structure‐based sequence alignment and for making high‐resolution figures. Sequence similarities and secondary structure details from aligned sequences were rendered using ESPript (Robert & Gouet, 2014). ESBRI (http://bioinformatica.isa.cnr.it/ESBRI/) was used to calculate salt bridges. PDBePISA (Krissinel, 2011) was used to determine the solvent‐accessible surface (SAS) area, interface area, and H‐bonds. ExPASy server (Gasteiger et al., 2005) was used to calculate the values of the charged residues. FASTA sequences and 3D PDB structures were downloaded from PDB (Burley et al., 2024). Molecular docking was carried out by employing the SwissDock web tool using the AutoDock Vina option (Grosdidier et al., 2011; Trott & Olson, 2010) with the p‐NPA as the ligand. The crystal structures of γ‐*Ca*CA and γ‐*Ca*CAmut were used as the receptors. Default parameters were employed for docking. The RING 4.0 web server platform was used in order to generate the residue interaction network and identify non‐covalent interactions at the protein structures (del Conte et al., 2024). The analysis was performed using the closest nodes and the multiple edges with a strict distance threshold (default platform options).

## AUTHOR CONTRIBUTIONS

**Charoutioun S. Bodourian:** Investigation; methodology; writing – review and editing; writing – original draft; data curation; formal analysis. **Mohsin Imran:** Investigation; writing – review and editing; formal analysis; writing – original draft; data curation. **Nikolaos D. Georgakis:** Investigation; writing – original draft; methodology; formal analysis; writing – review and editing; data curation. **Anastassios C. Papageorgiou:** Supervision; writing – review and editing; formal analysis; investigation; writing – original draft; data curation. **Nikolaos E. Labrou:** Conceptualization; writing – original draft; writing – review and editing; supervision; methodology; investigation.

## CONFLICT OF INTEREST STATEMENT

None of the authors have a conflict of interest to disclose.

## Supporting information


**FIGURE S1.** (a) SDS‐PAGE (12% w/v polyacrylamide) analysis of the purification of *γ‐Ca*CA by Ni‐NDA‐Sepharose affinity column. Lane 1: Protein markers; Lane 2: Crude *E. coli* Rosetta 2(DE3)pLysS lysate, expressing the recombinant *γ‐Ca*CA; Lane 3: Eluted fraction using 250 mM imidazole; Lane 4: Eluted fraction using 300 mM imidazole. (b) SDS‐PAGE (12% w/v polyacrylamide) analysis of the purification of *γ‐Ca*CAmut by Ni‐NDA‐Sepharose affinity column. Lane 1: Protein markers; Lane 2: Crude *E. coli* Rosetta 2(DE3)pLysS lysate, expressing the recombinant *γ‐Ca*CAmut; Lane 3: Eluted fraction using 300 mM imidazole.
**FIGURE S2.** CO_2_ hydration activity of γ‐CA variants. CO_2_ hydration activity measured by stopped‐flow assay at 0°C, expressed as Wilbur‐Anderson units per mg of enzyme protein for wild‐type γ‐*Ca*CA and mutant γ‐*Ca*CAmut. Data represent four independent experiments with at least three technical replicates each. Error bars indicate standard deviation. Statistical analysis (one‐tailed unpaired t‐test) comparing the reaction times of the enzymatically and non‐enzymatically catalyzed reactions yielded p‐values of 0.378 and 0.056 for the wild type and the mutant, respectively.
**FIGURE S3.** Mass photometry analysis of the oligomeric state of γ‐*Ca*CAmut at pH 8.0. The histogram shows the particle counts of γ‐*Ca*CAmut at the indicated molecular mass. The dark blue line is Gaussian fit to the peak. The calculated mass and its standard deviation (σ) are indicated above the peak. The peak corresponds to the trimeric enzyme structure.

**DATA S1.** Fluorescence raw DSF data for Figures 2 and 6e.

**DATA S2.** Activity measurements for pNPA hydrolysis by γ*Ca*CAmut enzyme, for Figure 6f.

## Data Availability

The data that support the findings of this study are available from the corresponding author upon reasonable request.

## References

[pro70396-bib-0001] Adams PD , Afonine PV , Bunkóczi G , Chen VB , Davis IW , Echols N , et al. PHENIX: a comprehensive python‐based system for macromolecular structure solution. Acta Crystallogr D Biol Crystallogr. 2010;66:213–221. 10.1107/S0907444909052925 20124702 PMC2815670

[pro70396-bib-0002] Alterio V , Langella E , De Simone G , Monti SM . Cadmium‐containing carbonic anhydrase CDCA1 in marine diatom Thalassiosira weissflogii. Mar Drugs. 2015;13:1688–1697. 10.3390/md13041688 25815892 PMC4413181

[pro70396-bib-0003] Altschul SF , Gish W , Miller W , Myers EW , Lipman DJ . Basic local alignment search tool. J Mol Biol. 1990;215:403–410. 10.1016/S0022-2836(05)80360-2 2231712

[pro70396-bib-0004] Angeli A . Bacterial γ‐carbonic anhydrases. Enzymes. Volume 55. Academic Press: Cambridge, Massachusetts; 2024. p. 93–120. 10.1016/bs.enz.2024.05.002 39223000

[pro70396-bib-0005] Apprill A , McNally S , Parsons R , Weber L . Minor revision to V4 region SSU rRNA 806R gene primer greatly increases detection of SAR11 bacterioplankton. Aquat Microb Ecol. 2015;75:129–137.

[pro70396-bib-0006] Arbab S , Ullah H , Khan MIU , Khattak MNK , Zhang J , Li K , et al. Diversity and distribution of thermophilic microorganisms and their applications in biotechnology. J Basic Microbiol. 2022;62:95–108. 10.1002/jobm.202100529 34878177

[pro70396-bib-0007] Aspatwar A , Tolvanen MEE , Ortutay C , Parkkila S . Carbonic anhydrase related proteins: molecular biology and evolution. Subcell Biochem. 2014;75:135–156. 10.1007/978-94-007-7359-2_8 24146378

[pro70396-bib-0008] Bokulich NA , Dillon MR , Bolyen E , Kaehler BD , Huttley GA , Caporaso JG . q2‐sample‐classifier: machine‐learning tools for microbiome classification and regression. J Open Source Softw. 2018;3(30):934. 10.21105/joss.00934 PMC6759219 31552137

[pro70396-bib-0009] Bolyen E , Rideout JR , Dillon MR , Bokulich NA , Abnet CC , Al‐Ghalith GA , et al. Reproducible, interactive, scalable and extensible microbiome data science using QIIME 2. Nat Biotechnol. 2019;37:852–857. 10.1038/s41587-019-0209-9 31341288 PMC7015180

[pro70396-bib-0010] Bunkóczi G , Read RJ . Improvement of molecular‐replacement models with sculptor. Acta Crystallogr D Biol Crystallogr. 2011;67:303–312. 10.1107/S0907444910051218 21460448 PMC3069745

[pro70396-bib-0011] Burkhardt C , Baruth L , Meyer‐Heydecke N , Klippel B , Margaryan A , Paloyan A , et al. Mining thermophiles for biotechnologically relevant enzymes: evaluating the potential of European and Caucasian hot springs. Extremophiles. 2023;28:5. 10.1007/s00792-023-01321-3 37991546 PMC10665251

[pro70396-bib-0012] Burley SK , Bhatt R , Bhikadiya C , Bi C , Biester A , Biswas P , et al. Updated resources for exploring experimentally‐determined PDB structures and computed structure models at the RCSB protein data Bank. Nucleic Acids Res. 2024;53:D564–D574. 10.1093/nar/gkae1091 PMC11701563 39607707

[pro70396-bib-0013] Callahan BJ , McMurdie PJ , Rosen MJ , Han AW , Johnson AJA , Holmes SP . DADA2: high‐resolution sample inference from Illumina amplicon data. Nat Methods. 2016;13:581–583. 10.1038/nmeth.3869 27214047 PMC4927377

[pro70396-bib-0014] Capasso C , de Luca V , Carginale V , Cannio R , Rossi M . Biochemical properties of a novel and highly thermostable bacterial α‐carbonic anhydrase from Sulfurihydrogenibium yellowstonense YO3AOP1. J Enzyme Inhib Med Chem. 2012;27:892–897. 10.3109/14756366.2012.703185 22803664

[pro70396-bib-0015] Capasso C , Luca VD , Carginale V , Caramuscio P , Cavalheiro C , Cannio R , et al. Characterization and properties of a new Thermoactive and thermostable carbonic anhydrase. Chem Eng Trans. 2012;27:271–276. 10.3303/CET1227046

[pro70396-bib-0016] Capasso C , Supuran CT . An overview of the alpha‐, beta‐ and gamma‐carbonic anhydrases from bacteria: can bacterial carbonic anhydrases shed new light on evolution of bacteria? J Enzyme Inhib Med Chem. 2015;30:325–332. 10.3109/14756366.2014.910202 24766661

[pro70396-bib-0017] Capasso C , Supuran CT . Bacterial ι‐CAs. Enzymes. 2024;55:121–142. 10.1016/bs.enz.2024.05.003 39222989

[pro70396-bib-0018] del Conte A , Camagni GF , Clementel D , Minervini G , Monzon AM , Ferrari C , et al. RING 4.0: faster residue interaction networks with novel interaction types across over 35,000 different chemical structures. Nucleic Acids Res. 2024;52:W306–W312. 10.1093/nar/gkae337 38686797 PMC11223866

[pro70396-bib-0019] del Prete S , Vullo D , Fisher GM , Andrews KT , Poulsen S‐A , Capasso C , et al. Discovery of a new family of carbonic anhydrases in the malaria pathogen *Plasmodium falciparum*: the η‐carbonic anhydrases. Bioorg Med Chem Lett. 2014;24:4389–4396. 10.1016/j.bmcl.2014.08.015 25168745

[pro70396-bib-0020] di Fiore A , de Luca V , Langella E , Nocentini A , Buonanno M , Monti SM , et al. Biochemical, structural, and computational studies of a γ‐carbonic anhydrase from the pathogenic bacterium Burkholderia pseudomallei. Comput Struct Biotechnol J. 2022;20:4185–4194. 10.1016/j.csbj.2022.07.033 36016712 PMC9389205

[pro70396-bib-0021] Emsley P , Cowtan K . Coot: model‐building tools for molecular graphics. Acta Crystallogr D Biol Crystallogr. 2004;60:2126–2132. 10.1107/S0907444904019158 15572765

[pro70396-bib-0022] Ferraroni M . Bacterial β‐carbonic anhydrases. Enzymes. Volume 55. Academic Press: Cambridge, Massachusetts; 2024. p. 65–91. 10.1016/bs.enz.2024.05.009 39222999

[pro70396-bib-0023] Fraser NJ , Liu J‐W , Mabbitt PD , Correy GJ , Coppin CW , Lethier M , et al. Evolution of protein quaternary structure in response to selective pressure for increased thermostability. J Mol Biol. 2016;428:2359–2371. 10.1016/j.jmb.2016.03.014 27016206

[pro70396-bib-0024] Fredslund F , Borchert MS , Poulsen J‐CN , Mortensen SB , Perner M , Streit WR , et al. Structure of a hyperthermostable carbonic anhydrase identified from an active hydrothermal vent chimney. Enzyme Microb Technol. 2018;114:48–54. 10.1016/j.enzmictec.2018.03.009 29685353

[pro70396-bib-0025] Fu Q , Kobayashi H , Kawaguchi H , Vilcaez J , Wakayama T , Maeda H , et al. Electrochemical and phylogenetic analyses of current‐generating microorganisms in a thermophilic microbial fuel cell. J Biosci Bioeng. 2013;115:268–271. 10.1016/j.jbiosc.2012.10.007 23164680

[pro70396-bib-0026] Fu X , Yu L‐J , Mao‐Teng L , Wei L , Wu C , Yun‐Feng M . Evolution of structure in gamma‐class carbonic anhydrase and structurally related proteins. Mol Phylogenet Evol. 2008;47:211–220. 10.1016/j.ympev.2008.01.005 18289884

[pro70396-bib-0027] Gasteiger E , Hoogland C , Gattiker A , Duvaud S , Wilkins MR , Appel RD , et al. Protein identification and analysis tools on the ExPASy server. In: Walker JM , editor. The proteomics protocols handbook. Totowa, NJ: Humana Press; 2005. p. 571–607. 10.1385/1-59259-890-0:571

[pro70396-bib-0028] Giovannelli D , Barry PH , de Moor JM , Jessen GL , Schrenk MO , Lloyd KG . Sampling across large‐scale geological gradients to study geosphere‐biosphere interactions. Front Microbiol. 2022;13:998133. 10.3389/fmicb.2022.998133 36386678 PMC9659755

[pro70396-bib-0029] Grosdidier A , Zoete V , Michielin O . SwissDock, a protein‐small molecule docking web service based on EADock DSS. Nucleic Acids Res. 2011;39:W270–W277. 10.1093/nar/gkr366 21624888 PMC3125772

[pro70396-bib-0030] Hebditch M , Carballo‐Amador MA , Charonis S , Curtis R , Warwicker J . Protein‐sol: a web tool for predicting protein solubility from sequence. Bioinformatics. 2017;33:3098–3100. 10.1093/bioinformatics/btx345 28575391 PMC5870856

[pro70396-bib-0031] Herlemann DP , Labrenz M , Jürgens K , Bertilsson S , Waniek JJ , Andersson AF . Transitions in bacterial communities along the 2000 km salinity gradient of the Baltic Sea. ISME J. 2011;5:1571–1579. 10.1038/ismej.2011.41 21472016 PMC3176514

[pro70396-bib-0032] Herrou J , Crosson S . Molecular structure of the Brucella abortus metalloprotein RicA, a Rab2‐binding virulence effector. Biochemistry. 2013;52:9020–9028. 10.1021/bi401373r 24251537 PMC3902126

[pro70396-bib-0033] Hirakawa Y , Hanawa Y , Yoneda K , Suzuki I . Evolution of a chimeric mitochondrial carbonic anhydrase through gene fusion in a haptophyte alga. FEBS Lett. 2022;596:3051–3059. 10.1002/1873-3468.14475 35997667

[pro70396-bib-0034] Huynh K , Partch CL . Analysis of protein stability and ligand interactions by thermal shift assay. Curr Protoc Protein Sci. 2015;79:28.9.1–28.9.14. 10.1002/0471140864.ps2809s79 PMC4332540 25640896

[pro70396-bib-0035] Jo BH , Seo JH , Cha HJ . Bacterial extremo‐α‐carbonic anhydrases from deep‐sea hydrothermal vents as potential biocatalysts for CO2 sequestration. J Mol Catal B: Enzym. 2014;109:31–39. 10.1016/j.molcatb.2014.08.002

[pro70396-bib-0036] Kielkopf CL , Bauer W , Urbatsch IL . Bradford assay for determining protein concentration. Cold Spring Harb Protoc. 2020;2020:102269. 10.1101/pdb.prot102269 32238597

[pro70396-bib-0037] Kisker C , Schindelin H , Alber BE , Ferry JG , Rees DC . A left‐hand beta‐helix revealed by the crystal structure of a carbonic anhydrase from the archaeon Methanosarcina thermophila. EMBO J. 1996;15:2323–2330. 8665839 PMC450161

[pro70396-bib-0038] Klindworth A , Pruesse E , Schweer T , Peplies J , Quast C , Horn M , et al. Evaluation of general 16S ribosomal RNA gene PCR primers for classical and next‐generation sequencing‐based diversity studies. Nucleic Acids Res. 2013;41:e1. 10.1093/nar/gks808 22933715 PMC3592464

[pro70396-bib-0039] Krissinel E . Macromolecular complexes in crystals and solutions. Acta Crystallogr D Biol Crystallogr. 2011;67:376–385. 10.1107/S0907444911007232 21460456 PMC3069753

[pro70396-bib-0040] Krissinel E , Henrick K . Secondary‐structure matching (SSM), a new tool for fast protein structure alignment in three dimensions. Acta Crystallogr D Biol Crystallogr. 2004;60:2256–2268. 10.1107/S0907444904026460 15572779

[pro70396-bib-0041] Laemmli UK . Cleavage of structural proteins during the assembly of the head of bacteriophage T4. Nature. 1970;227:680–685. 10.1038/227680a0 5432063

[pro70396-bib-0042] Lambrakis N , Katsanou K , Siavalas G . Geothermal fields and thermal waters of Greece: an overview. In: Baba A , Bundschuh J , Chandrasekharam D , editors. Geothermal systems and energy resources: Turkey and Greece. Volume 7.CRC Press: Florida; 2013. p. 25–46.

[pro70396-bib-0043] Lionetto MG , Caricato R , Giordano ME , Schettino T . The complex relationship between metals and carbonic anhydrase: new insights and perspectives. Int J Mol Sci. 2016;17:127. 10.3390/ijms17010127 26797606 PMC4730368

[pro70396-bib-0044] Liu R , Qiao X , Shi Y , Peterson CB , Bush WS , Cominelli F , et al. Constructing phylogenetic trees for microbiome data analysis: a mini‐review. Comput Struct Biotechnol J. 2024;23:3859–3868. 10.1016/j.csbj.2024.10.032 39554614 PMC11564040

[pro70396-bib-0045] Ludwig M . Evolution of carbonic anhydrase in C4 plants. Curr Opin Plant Biol. 2016;31:16–22. 10.1016/j.pbi.2016.03.003 27016649

[pro70396-bib-0046] Macauley SR , Zimmerman SA , Apolinario EE , Evilia C , Hou Y‐M , Ferry JG , et al. The archetype gamma‐class carbonic anhydrase (cam) contains iron when synthesized in vivo. Biochemistry. 2009;48:817–819. 10.1021/bi802246s 19187031

[pro70396-bib-0047] Matthews BW . Solvent content of protein crystals. J Mol Biol. 1968;33:491–497. 10.1016/0022-2836(68)90205-2 5700707

[pro70396-bib-0048] McCoy AJ , Grosse‐Kunstleve RW , Adams PD , Winn MD , Storoni LC , Read RJ . Phaser crystallographic software. J Appl Cryst. 2007;40:658–674. 10.1107/S0021889807021206 19461840 PMC2483472

[pro70396-bib-0049] Mesbah NM . Industrial biotechnology based on enzymes from extreme environments. Front Bioeng Biotechnol. 2022;10:870083. 10.3389/fbioe.2022.870083 35480975 PMC9036996

[pro70396-bib-0050] Mikulski RL , Silverman DN . Proton transfer in catalysis and the role of proton shuttles in carbonic anhydrase. Biochim Biophys Acta. 2010;1804:422–426. 10.1016/j.bbapap.2009.08.003 19679199 PMC2818086

[pro70396-bib-0051] Nocentini A , Supuran CT , Capasso C . An overview on the recently discovered iota‐carbonic anhydrases. J Enzyme Inhib Med Chem. 2021;36:1988–1995. 10.1080/14756366.2021.1972995 34482770 PMC8425729

[pro70396-bib-0052] Ogg CD , Patel BKC . Caloramator australicus sp. nov., a thermophilic, anaerobic bacterium from the great Artesian Basin of Australia. Int J Syst Evol Microbiol. 2009;59:95–101. 10.1099/ijs.0.000802-0 19126731

[pro70396-bib-0053] Ogg CD , Patel BKC . Draft genome sequence of Caloramator australicus strain RC3T, a thermoanaerobe from the great Artesian Basin of Australia. J Bacteriol. 2011;193:2664–2665. 10.1128/JB.00193-11 21421756 PMC3133146

[pro70396-bib-0054] Pantiora PD , Georgakis ND , Premetis GE , Labrou NE . Metagenomic analysis of hot spring soil for mining a novel thermostable enzybiotic. Appl Microbiol Biotechnol. 2024;108:163. 10.1007/s00253-023-12979-2 38252132 PMC10803476

[pro70396-bib-0055] Parisi G , Fornasari M , Echave J . Evolutionary analysis of gamma‐carbonic anhydrase and structurally related proteins. Mol Phylogenet Evol. 2000;14:323–334. 10.1006/mpev.1999.0734 10712838

[pro70396-bib-0056] Park H‐M , Park J‐H , Choi J‐W , Lee J , Kim BY , Jung C‐H , et al. Structures of the γ‐class carbonic anhydrase homologue YrdA suggest a possible allosteric switch. Acta Crystallogr D Biol Crystallogr. 2012;68:920–926. 10.1107/S0907444912017210 22868757

[pro70396-bib-0057] Pedregosa F , Varoquaux G , Gramfort A , Michel V , Thirion B , Grisel O , et al. Scikit‐ learn: machine learning in python. J Mach Learn Res. 2012;12: 2825‐2830.

[pro70396-bib-0058] Pettersen EF , Goddard TD , Huang CC , Couch GS , Greenblatt DM , Meng EC , et al. UCSF chimera‐‐a visualization system for exploratory research and analysis. J Comput Chem. 2004;25:1605–1612. 10.1002/jcc.20084 15264254

[pro70396-bib-0059] Podar PT , Yang Z , Björnsdóttir SH , Podar M . Comparative analysis of microbial diversity across temperature gradients in Hot Springs from Yellowstone and Iceland. Front Microbiol. 2020;11:1625. 10.3389/fmicb.2020.01625 32760379 PMC7372906

[pro70396-bib-0060] Qing R , Hao S , Smorodina E , Jin D , Zalevsky A , Zhang S . Protein design: from the aspect of water solubility and stability. Chem Rev. 2022;122:14085–14179. 10.1021/acs.chemrev.1c00757 35921495 PMC9523718

[pro70396-bib-0061] Quast C , Pruesse E , Yilmaz P , Gerken J , Schweer T , Yarza P , et al. The SILVA ribosomal RNA gene database project: improved data processing and web‐based tools. Nucleic Acids Res. 2013;41:D590–D596. 10.1093/nar/gks1219 23193283 PMC3531112

[pro70396-bib-0062] Regueira‐Iglesias A , Balsa‐Castro C , Blanco‐Pintos T , Tomás I . Critical review of 16S rRNA gene sequencing workflow in microbiome studies: from primer selection to advanced data analysis. Mol Oral Microbiol. 2023;38:347–399. 10.1111/omi.12434 37804481

[pro70396-bib-0063] Robert X , Gouet P . Deciphering key features in protein structures with the new ENDscript server. Nucleic Acids Res. 2014;42:W320–W324. 10.1093/nar/gku316 24753421 PMC4086106

[pro70396-bib-0064] Salmaso N , Albanese D , Capelli C , Boscaini A , Pindo M , Donati C . Diversity and cyclical seasonal transitions in the bacterial Community in a Large and Deep Perialpine Lake. Microb Ecol. 2018;76:125–143. 10.1007/s00248-017-1120-x 29192335

[pro70396-bib-0065] Smith KS , Ferry JG . A plant‐type (β‐class) carbonic anhydrase in the thermophilic Methanoarchaeon Methanobacterium thermoautotrophicum. J Bacteriol. 1999;181:6247–6253. 10.1128/jb.181.20.6247-6253.1999 10515911 PMC103756

[pro70396-bib-0066] Sridharan U , Ragunathan P , Kuramitsu S , Yokoyama S , Kumarevel T , Ponnuraj K . Structural and functional characterization of a putative carbonic anhydrase from Geobacillus kaustophilus reveals its cambialistic function. Biochem Biophys Res Commun. 2021;547:96–101. 10.1016/j.bbrc.2021.02.036 33610046

[pro70396-bib-0067] Supuran CT . Structure and function of carbonic anhydrases. Biochem J. 2016a;473:2023–2032. 10.1042/BCJ20160115 27407171

[pro70396-bib-0068] Supuran CT . How many carbonic anhydrase inhibition mechanisms exist? J Enzyme Inhib Med Chem. 2016b;31:345–360. 10.3109/14756366.2015.1122001 26619898

[pro70396-bib-0069] Supuran CT . A simple yet multifaceted 90 years old, evergreen enzyme: carbonic anhydrase, its inhibition and activation. Bioorg Med Chem Lett. 2023;93:129411. 10.1016/j.bmcl.2023.129411 37507055

[pro70396-bib-0070] Thompson JD , Higgins DG , Gibson TJ . CLUSTAL W: improving the sensitivity of progressive multiple sequence alignment through sequence weighting, position‐specific gap penalties and weight matrix choice. Nucleic Acids Res. 1994;22:4673–4680. 10.1093/nar/22.22.4673 7984417 PMC308517

[pro70396-bib-0071] Trott O , Olson AJ . AutoDock Vina: improving the speed and accuracy of docking with a new scoring function, efficient optimization, and multithreading. J Comput Chem. 2010;31:455–461. 10.1002/jcc.21334 19499576 PMC3041641

[pro70396-bib-0072] Verpoorte JA , Mehta S , Edsall JT . Esterase activities of human carbonic anhydrases B and C. J Biol Chem. 1967;242:4221–4229. 4964830

[pro70396-bib-0073] Wang W , Zhang Y , Wang L , Jing Q , Wang X , Xi X , et al. Molecular structure of thermostable and zinc‐ion‐binding γ‐class carbonic anhydrases. Biometals. 2019;32:317–328. 10.1007/s10534-019-00190-8 30895492

[pro70396-bib-0074] Weiss S , Xu ZZ , Peddada S , Amir A , Bittinger K , Gonzalez A , et al. Normalization and microbial differential abundance strategies depend upon data characteristics. Microbiome. 2017;5:27. 10.1186/s40168-017-0237-y 28253908 PMC5335496

[pro70396-bib-0075] Williams AD , Leung VW , Tang JW , Hidekazu N , Suzuki N , Clarke AC , et al. Ancient environmental microbiomes and the cryosphere. Trends Microbiol. 2024;33:233–249. 10.1016/j.tim.2024.09.010 39487079

[pro70396-bib-0076] Williams CJ , Headd JJ , Moriarty NW , Prisant MG , Videau LL , Deis LN , et al. MolProbity: more and better reference data for improved all‐atom structure validation. Protein Sci. 2018;27:293–315. 10.1002/pro.3330 29067766 PMC5734394

[pro70396-bib-0077] Yilmaz P , Parfrey LW , Yarza P , Gerken J , Pruesse E , Quast C , et al. The SILVA and “all‐species living tree project (LTP)” taxonomic frameworks. Nucleic Acids Res. 2014;42:D643–D648. 10.1093/nar/gkt1209 24293649 PMC3965112

[pro70396-bib-0078] Zimmerman SA , Tomb J‐F , Ferry JG . Characterization of CamH from Methanosarcina thermophila, founding member of a subclass of the {gamma} class of carbonic anhydrases. J Bacteriol. 2010;192:1353–1360. 10.1128/JB.01164-09 20023030 PMC2820857

