# Supplementary material for: Structural and functional characterization of a metagenomically derived γ‐type carbonic anhydrase and its engineering into a hyperthermostable esterase
Source: Protein Sci. 2025 Nov 26;34(12):e70396. doi: 10.1002/pro.70396 (PMC12648627; doi:10.1002/pro.70396)
Supplement: Supplementary file 1 — FIGURE S1. (a) SDS‐PAGE (12% w/v polyacrylamide) analysis of the purification of γ‐CaCA by Ni‐NDA‐Sepharose affinity column. Lane 1: Protein markers; Lane 2: Crude E. coli Rosetta 2(DE3)pLysS lysate, expressing the recombinant γ‐CaCA; Lane 3: Eluted fraction using 250 mM imidazole; Lane 4: Eluted fraction using 300 mM imidazole. (b) SDS‐PAGE (12% w/v polyacrylamide) analysis of the purification of γ‐CaCAmut by Ni‐NDA‐Sepharose affinity column. Lane 1: Protein markers; Lane 2: Crude E. coli Rosetta 2(DE3)pLysS lysate, expressing the recombinant γ‐CaCAmut; Lane 3: Eluted fraction using 300 mM imidazole. FIGURE S2. CO2 hydration activity of γ‐CA variants. CO2 hydration activity measured by stopped‐flow assay at 0°C, expressed as Wilbur‐Anderson units per mg of enzyme protein for wild‐type γ‐CaCA and mutant γ‐CaCAmut. Data represent four independent experiments with at least three technical replicates each. Error bars indicate standard deviation. Statistical analysis (one‐tailed unpaired t‐test) comparing the reaction times of the enzymatically and non‐enzymatically catalyzed reactions yielded p‐values of 0.378 and 0.056 for the wild type and the mutant, respectively. FIGURE S3. Mass photometry analysis of the oligomeric state of γ‐CaCAmut at pH 8.0. The histogram shows the particle counts of γ‐CaCAmut at the indicated molecular mass. The dark blue line is Gaussian fit to the peak. The calculated mass and its standard deviation (σ) are indicated above the peak. The peak corresponds to the trimeric enzyme structure. [file PRO-34-e70396-s002.docx]

| **A**  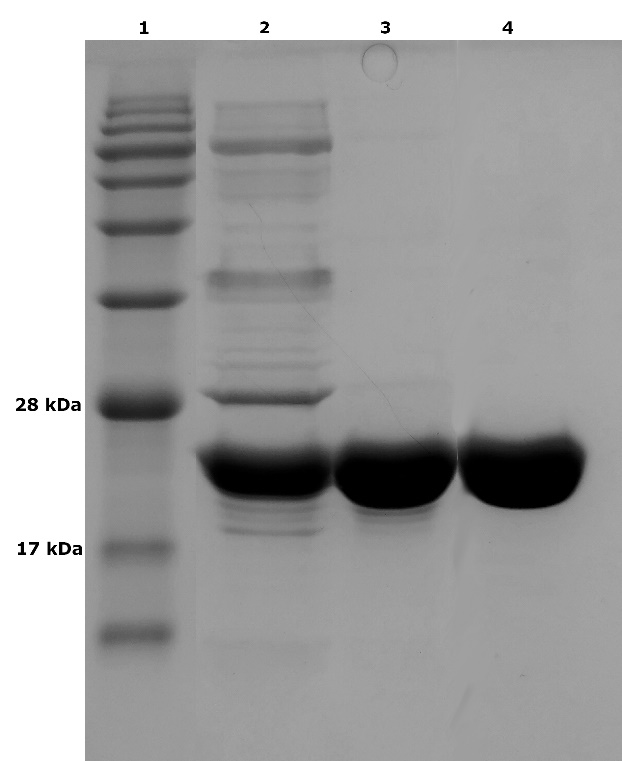 | **B**  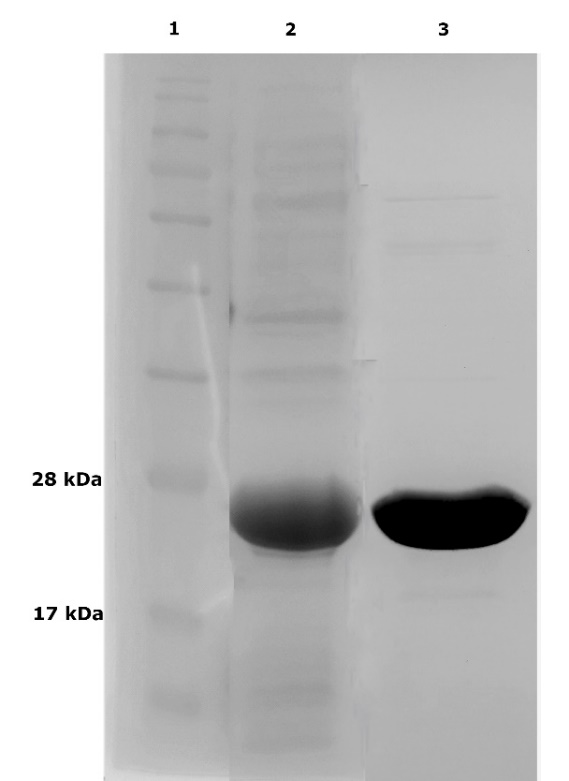 |
| --- | --- |

**Figure S1.** **A:** SDS-PAGE (12% w/v polyacrylamide) analysis of the purification of *γ-Ca*CA by Ni-NDA-Sepharose affinity column. Lane 1: Protein markers; Lane 2: Crude *E. coli* Rosetta 2(DE3)pLysS lysate, expressing the recombinant *γ-Ca*CA; Lane 3: Eluted fraction using 250 mM imidazole; Lane 4: Eluted fraction using 300 mM imidazole. **B:** SDS-PAGE (12% w/v polyacrylamide) analysis of the purification of *γ-Ca*CAmut by Ni-NDA-Sepharose affinity column. Lane 1: Protein markers; Lane 2: Crude *E. coli* Rosetta 2(DE3)pLysS lysate, expressing the recombinant *γ-Ca*CAmut; Lane 3: Eluted fraction using 300 mM imidazole.


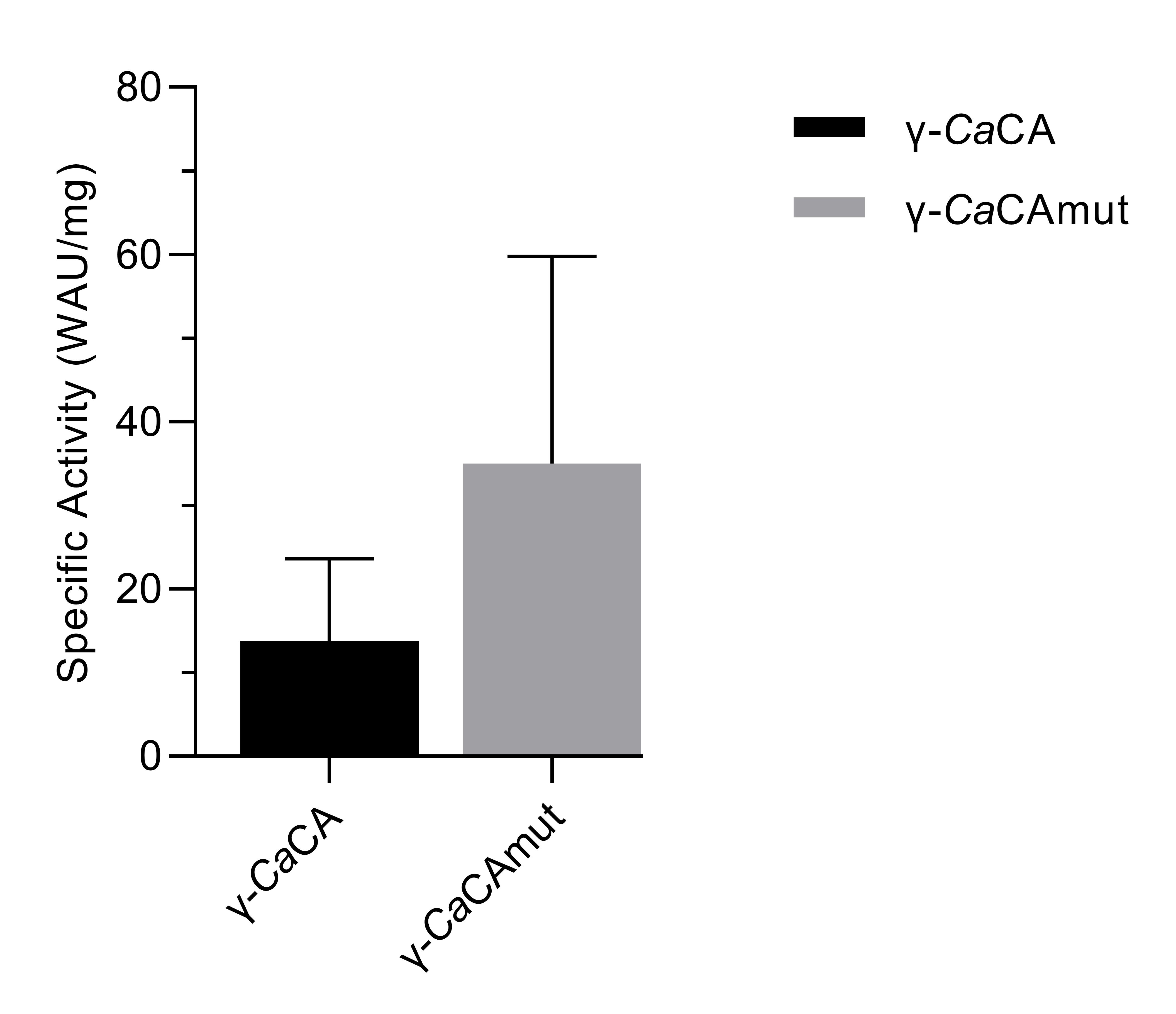


**Figure S2**. CO₂ hydration activity of γ-CA variants. CO₂ hydration activity measured by stopped-flow assay at 0°C, expressed as Wilbur-Anderson units per mg of enzyme protein for wild-type γ-*Ca*CA and mutant γ-*Ca*CAmut. Data represent four independent experiments with at least three technical replicates each. Error bars indicate standard deviation. Statistical analysis (one-tailed unpaired t-test) comparing the reaction times of the enzymatically and non-enzymatically catalyzed reactions yielded p-values of 0.378 and 0.056 for the wild type and the mutant, respectively.


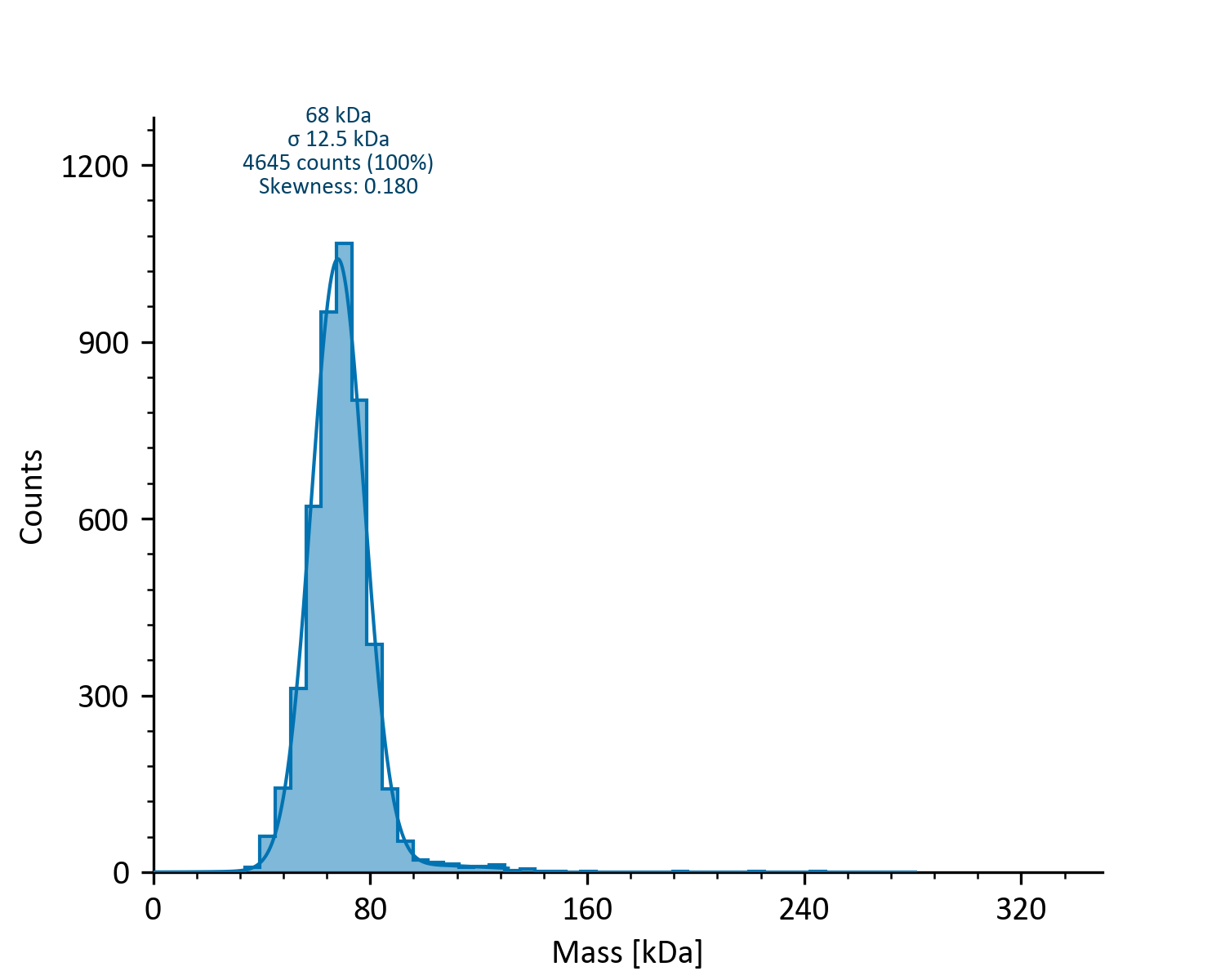


**Figure S3.** Mass photometry analysis of the oligomeric state of γ-*Ca*CAmut at pH 8.0. The histogram shows the particle counts of γ-*Ca*CAmut at the indicated molecular mass. The dark blue line is Gaussian fit to the peak. The calculated mass and its standard deviation (σ) are indicated above the peak. The peak corresponds to the trimeric enzyme structure.
